# Ferritinophagy Rewires Carnitine‐Dependent Lipid Metabolism to Inhibit PRRSV and IAV Replication

**DOI:** 10.1002/advs.75721

**Published:** 2026-05-20

**Authors:** Kaifeng Guan, Chenyang Yuan, Zekun Meng, Yanan Wang, Xueying Zhai, Chunjie Huang, Xiang Zhou, Gaiping Zhang

**Affiliations:** ^1^ School of Advanced Agricultural Sciences Peking University Beijing China; ^2^ Longhu Laboratory Zhengzhou University Zhengzhou China; ^3^ College of Veterinary Medicine Northwest A&F University Yangling China; ^4^ International Joint Research Center of National Animal Immunology College of Veterinary Medicine Henan Agricultural University Zhengzhou China; ^5^ Institute of Reproductive Medicine School of Medicine Nantong University Nantong China; ^6^ Hubei Hongshan Laboratory College of Animal Science and Technology Huazhong Agricultural University Wuhan China; ^7^ Institute for Animal Health (Key Laboratory of Animal Immunology) Henan Academy of Agricultural Sciences Zhengzhou China

**Keywords:** carnitine, ferritinophagy, influenza A virus​, iron‐sulfur cluster​s, lipid metabolism, NCOA4, PRRSV

## Abstract

Ferritinophagy is crucial for maintaining iron homeostasis and regulating iron‐dependent viral replication. This study demonstrates that ferritinophagy reprograms carnitine‐dependent lipid metabolism by impairing Fe‐S clusters biogenesis, thereby inhibiting iron‐dependent viral replication. Specifically, NCOA4‐mediated ferritinophagy disrupts Fe‐S clusters assembly through the autophagic degradation of MMS19, leading to mitochondrial metabolic remodeling and suppression of carnitine biosynthesis. Carnitine deficiency subsequently destabilizes the proteins TMED10, HDLBP, and RAB40C via specific amino acid residues (Asp78, Leu336, and Glu154, respectively), and we reveal that carnitine directly stabilizes these lipid droplet‐associated proteins, promoting lipid droplet formation. Collectively, these changes orchestrate an iron‐lipid metabolic axis that inhibits the replication of diverse PRRSV strains as well as influenza A virus (IAV). Conversely, PRRSV counteracts this antiviral mechanism by promoting autophagic degradation of NCOA4 via K63‐linked ubiquitination, a process in which the viral protein Nsp5 recruits the E3 ligase adaptor DDB1 to mediate ubiquitination. Our findings establish NCOA4 as a link between ferritinophagy and lipid metabolic reprogramming, revealing a novel antiviral pathway and providing foundational insights for developing innovative antiviral strategies.

## Graphical abstract

NCOA4‐mediated ferritinophagy reprograms carnitine metabolism by disrupting Fe‐S cluster biogenesis, thereby establishing an iron‐lipid axis that suppresses various viruses, including PRRSV and IAV. However, viruses counteract this mechanism by degrading NCOA4. This study reveals a novel metabolic antiviral pathway that connects ferritinophagy with lipid remodeling.

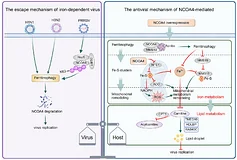

## 1 Introduction

Host‐virus interactions fundamentally shape antiviral immunity. Viruses exploit iron‐dependent enzymes such as RNA‐dependent RNA polymerase (RdRp) and ribonucleotide reductase (RNR) to hijack host iron metabolism in order to facilitate their replication [1, 2, 3]. These iron‐dependent viruses, including porcine reproductive and respiratory syndrome virus (PRRSV) and influenza virus depend on intracellular iron homeostasis for efficient propagation [4, 5]. Recent research has demonstrated that disruption of host iron metabolism can markedly inhibit viral replication [6, 7, 8]. However, the precise molecular mechanisms underlying this phenomenon remain unclear. Ferritinophagy, a selective autophagic process that degrades the iron‐storage protein ferritin through the cargo receptor NCOA4, plays a critical role in maintaining iron homeostasis and modulating viral replication [9, 10].

Similarly, lipid metabolic reprogramming is pivotal for viral replication. Viruses hijack host lipid metabolism to generate lipid droplets (LDs), which provide essential lipids for viral membrane formation and supply energy for their replication [11, 12]. Conversely, LDs can also suppress viral replication through metabolic competition, autophagy, and oxidative stress responses [11, 12, 13]. An emerging interplay between iron and lipid metabolism has been documented in other contexts [14]. Nevertheless, how these two metabolic pathways are integrated to coordinate an antiviral response remains undefined. Specifically, it remains unclear whether and by what mechanisms ferritinophagy influences lipid metabolism, as well as how viruses may directly manipulate the ferritinophagy pathway to facilitate immune evasion. Thus, elucidating how NCOA4‐mediated ferritinophagy inhibits iron‐dependent viruses through an iron‐lipid metabolism axis is crucial for developing targeted antiviral strategies. Here, we propose the “iron‐lipid metabolism axis” as a conceptual framework describing the coordinated crosstalk wherein cellular iron homeostasis (governed by ferritinophagy) directly regulates lipid metabolic reprogramming, and the dynamic balance of this axis determines the host's permissiveness to viral replication.

PRRSV serves as a valuable model for investigating iron‐dependent viral mechanisms due to its reliance on iron ions and its association with mitochondrial remodeling [4, 15]. Notably, overexpression of the PRRSV nucleocapsid (N) protein leads to the downregulation of host iron transport proteins and ferritin heavy chain 1 (FTH1) [16]. Additionally, both induction of autophagy and iron modulation exhibit anti‐PRRSV activity [17, 18]. Metabolomic analyses reveal altered lipid metabolism in PRRSV‐infected cells [19], yet its link to iron metabolic pathways remains unclear. Iron‐sulfur clusters, composed of iron ions and sulfide ions, are ubiquitous cofactors that play a critical role in viral replication [20, 21]. Disrupting iron‐sulfur clusters inhibits viral replication, a process mediated by the key factor MMS19 [2, 22]. Critically, it remains unresolved how NCOA4 influences viral replication through the ferritinophagy pathway and whether additional regulatory mechanisms beyond iron metabolism are involved. Accordingly, a principal objective of the present study is to investigate whether NCOA4‐mediated ferritinophagy affects iron‐dependent viral replication by disrupting iron‐sulfur clusters, thereby modulating the cellular iron‐lipid metabolic axis.

This study demonstrates that NCOA4 facilitates ferritinophagy‐dependent degradation of MMS19, thereby impairing iron‐sulfur clusters biogenesis and inducing mitochondrial metabolic remodeling. These changes lead to diminished Fe^2^^+^ availability and suppression of carnitine biosynthesis, which in turn trigger the destabilization of TMED10, HDLBP, and RAB40C—proteins identified through molecular docking and limited proteolysis–mass spectrometry (LiP‐SMap) analysis of carnitine interactors. We further demonstrate that carnitine directly stabilizes these lipid droplet regulatory proteins. Concurrently, metabolic reprogramming promotes lipid droplet formation. Collectively, these molecular and metabolic alterations inhibit the replication of diverse PRRSV strains as well as IAV. Conversely, the viral countermeasure involves PRRSV‐encoded Nsp5, which subverts this pathway by recruiting the E3 ligase adaptor DDB1 to promote autophagic degradation of NCOA4 through K63‐linked ubiquitination, thereby facilitating viral propagation. This study introduces an innovative iron‐lipid metabolism axis model, providing a mechanistic framework for targeting metabolism in antiviral strategies.

## 2 Results

### 2.1 PRRSV Infection Reduces NCOA4 Expression

**FIGURE 1:**
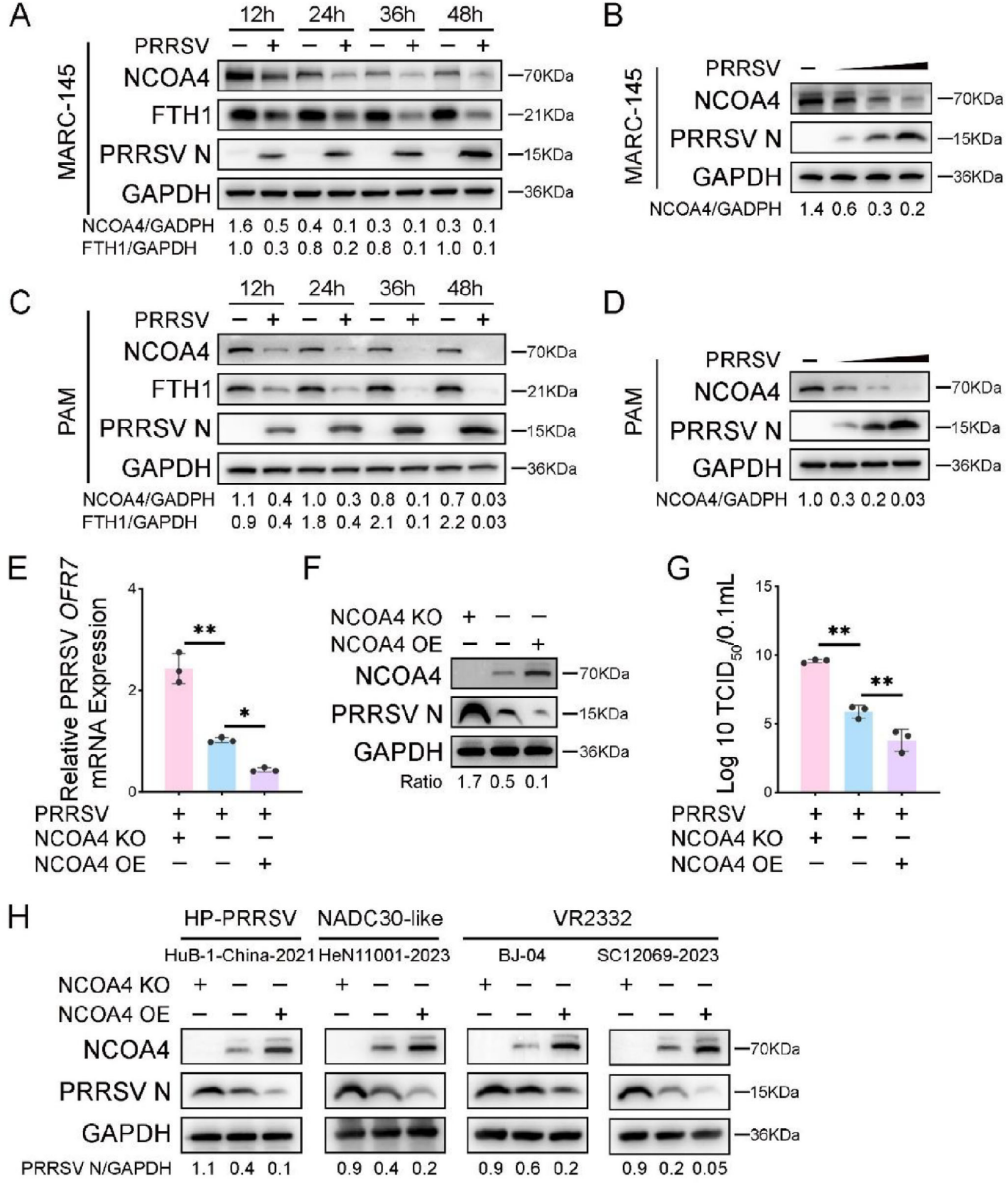
PRRSV infection inhibits NCOA4 expression. (A) (C)MARC‐145 (A) or PAM cells (C) were infected with PRRSV (MOI = 1) and harvested at the indicated time points post‐infection (12, 24, 36, 48 h). Immunoblotting was conducted to assess the levels of NCOA4, FTH1, and PRRSV N proteins. (B) (D) MARC‐145 (B) or PAM cells (D) were infected with PRRSV at various MOIs (0.5MOI, 1MOI, and 5MOI), and cells were collected 24 h post‐infection. Immunoblotting was performed to evaluate the expression of NCOA4 and PRRSV N proteins. (Original blot images in Figure S9 1a‐1d). (E) Wild‐type (WT) MARC‐145 cells were transfected to overexpress either pcDNA3.1‐NCOA4 or an empty pcDNA3.1 vector, while NCOA4 knockout MARC‐145 cell lines were infected with PRRSV at MOI = 1. Cells were collected 24 h post‐infection, and RT‐qPCR was utilized to quantify *ORF7* mRNA levels, normalized to the reference gene GAPDH. ^*^ *p* < 0.05, ^**^ *p* < 0.01, one‐way ANOVA, *n* = 3. (F) Immunoblotting was conducted to analyze the expression of NCOA4 and PRRSV N proteins, following the same treatment as in (E). (Original blot images in Figure S9 1e). (G) The TCID_50_ method was employed to assess viral titers. PRRSV was propagated in NCOA4 KO, NCOA4 OE, and WT MARC‐145 cells, and the virus was collected and diluted in a tenfold series to infect WT MARC‐145 cells. Cytopathic effects were monitored, and viral titers were calculated using the Karber method. ^**^ *p* < 0.01, one‐way ANOVA, *n* = 3. (H) NCOA4 KO, NCOA4 OE, and WT MARC‐145 cells were infected with HP‐PRRSV (HuB‐1‐China‐2021), NADC30‐like (HeN11001‐2023), and VR2332 (BJ‐04, SC12069‐2023). Cells were harvested 24 h post‐infection, and immunoblotting was performed to detect the expression of PRRSV N protein. (Original blot images in Figure S9 1f).

To investigate the connection between NCOA4 and PRRSV, MARC‐145 and porcine alveolar macrophage (PAM) cells were infected with the HP‐PRRSV strain (HeN07), respectively. Immunoblotting analysis revealed that PRRSV infection significantly reduced NCOA4 expression in MARC‐145 and PAM cells in a time‐ and dose‐dependent manner (Figure 1A–D; Figure S1A–D). To determine the functional significance of this downregulation, we generated a stable *NCOA4*‐knockout MARC‐145 cell line using CRISPR‐Cas9. The results showed that knocking out NCOA4 greatly enhanced the replication of PRRSV (HeN07) at the mRNA, protein, and TCID_50_ levels, whereas overexpressing NCOA4 produced contrary effects (Figure 1E–G; Figure S1E). Furthermore, infection with diverse PRRSV strains (classical, highly pathogenic, and NADC30‐like) consistently downregulated NCOA4 protein expression (Figure S1G,H), and NCOA4 overexpression broadly inhibited the replication of these strains (Figure 1H; Figure S1F). These results identify NCOA4 as a host factor that restricts PRRSV infection.

### 2.2 NCOA4 Inhibits PRRSV Replication via Mitochondrial Remodeling

**FIGURE 2:**
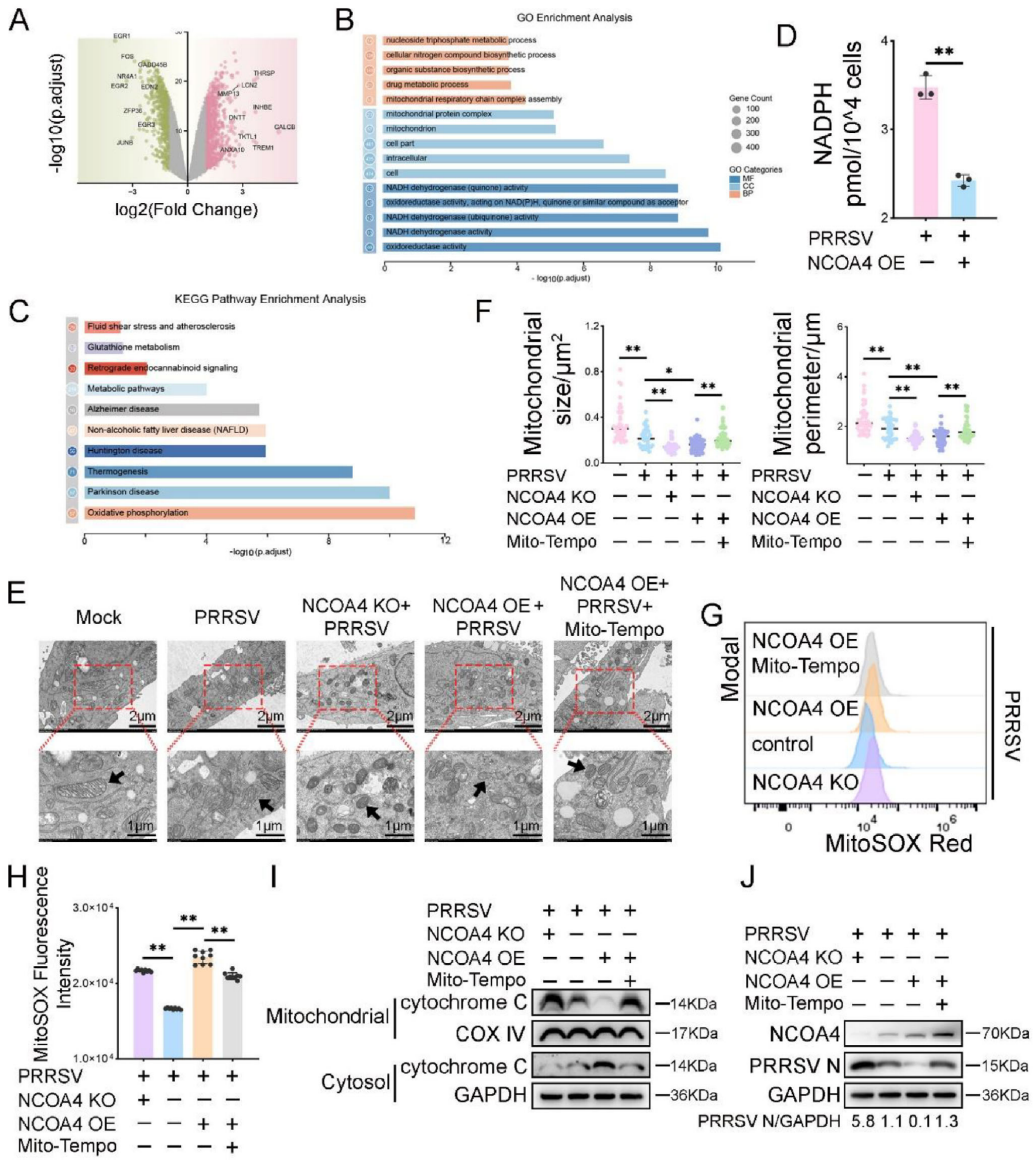
NCOA4 inhibits PRRSV replication via mitochondrial remodeling. (A–C) RNA sequencing was performed on MARC‐145 cells following NCOA4 overexpression and PRRSV infection. (A) Volcano plot displaying differentially expressed genes; (B) Gene Ontology (GO) analysis; (C) Kyoto Encyclopedia of Genes and Genomes (KEGG) pathway analysis. (D) NADPH levels were measured 24 h after PRRSV infection in MARC‐145 cells with NCOA4 overexpression, reported as pmol/10^4 cells. ^**^ *p* < 0.01, Student's *t*‐test, *n* = 3. (E) PRRSV was introduced to NCOA4 KO, NCOA4 OE, and WT MARC‐145 cells (MOI = 1), with a mock group as a negative control. Cells were harvested for observation by transmission electron microscopy (TEM) imaging. (F) Statistical analysis of the size and perimeter of mitochondria across different treatments. ^*^ *p* < 0.05, ^**^ *p* < 0.01, one‐way ANOVA, *n* ≥ 3. (G) Mitochondrial ROS levels were assessed using flow cytometry, with the same cell treatments as previously mentioned. (H) Quantitative analysis of the average fluorescence intensity of MitoSOX Red. ^**^ *p* < 0.01, one‐way ANOVA, *n* ≥ 3. (I) Immunoblotting analysis was conducted to assess cytochrome C release, using the same cell treatments. (Original blot images in Figure S9 2a). (J) Immunoblotting analysis was also performed to evaluate the expression of NCOA4 and the PRRSV N protein, with consistent cell treatments. (Original blot images in Figure S9 2b).

To elucidate the mechanism by which NCOA4 inhibits PRRSV, we performed transcriptome sequencing on PRRSV‐infected MARC‐145 cells with or without NCOA4 overexpression (Figure S2A). The results showed that a total of 1888 differentially expressed genes (DEGs) were identified, of which 1070 genes were upregulated and 818 genes were downregulated (fold change >1.5 and *p* < 0.05) (Figure 2A; Figure S2B). Functional enrichment and KEGG pathway analysis indicated that these DEGs were predominantly associated with NADH and mitochondrial functions (Figure 2B), with metabolic pathways showing the highest gene enrichment (Figure 2C). This suggests that NCOA4 overexpression may inhibit PRRSV replication via mitochondrial metabolic pathways. Consistent with this, NCOA4 overexpression significantly reduced intracellular NADPH levels (Figure 2D), pointing to an impact on mitochondrial respiration and energy metabolism. To further investigate how NCOA4 affects mitochondria, Mito‐Tempo (a mitochondrial reductant) was introduced to restore normal mitochondrial function. Transmission electron microscopy revealed that NCOA4 overexpression caused significant mitochondrial remodeling (including size and perimeter), which Mito‐Tempo alleviated (Figure 2E,F). Flow cytometry showed that NCOA4 overexpression increased mitochondrial oxidative stress, while NCOA4 knockout had the opposite effect; Mito‐Tempo treatment significantly reduced oxidative stress levels (Figure 2G,H). Immunoblotting of separated cytoplasm and mitochondria indicated that NCOA4 overexpression led to downregulation of cytochrome C in mitochondria and upregulation in the cytoplasm, which Mito‐Tempo could restore, suggesting that NCOA4 promotes mitochondrial remodeling that results in cytochrome C release into the cytoplasm (Figure 2I). Additionally, immunoblotting showed that NCOA4 inhibits PRRSV replication, and this inhibition was reduced with Mito‐Tempo treatment (Figure 2J; Figure S2C). These findings imply that NCOA4 inhibits PRRSV replication through mitochondrial pathways rather than immune pathways, as NCOA4 overexpression significantly reduced TNF‐α levels (Figure S2D) but did not affect p65, p‐p65, or STING protein levels (Figure S2E).

### 2.3 NCOA4‐Mediated Ferritinophagy Disrupts Iron‐Sulfur Clusters Biogenesis

**FIGURE 3:**
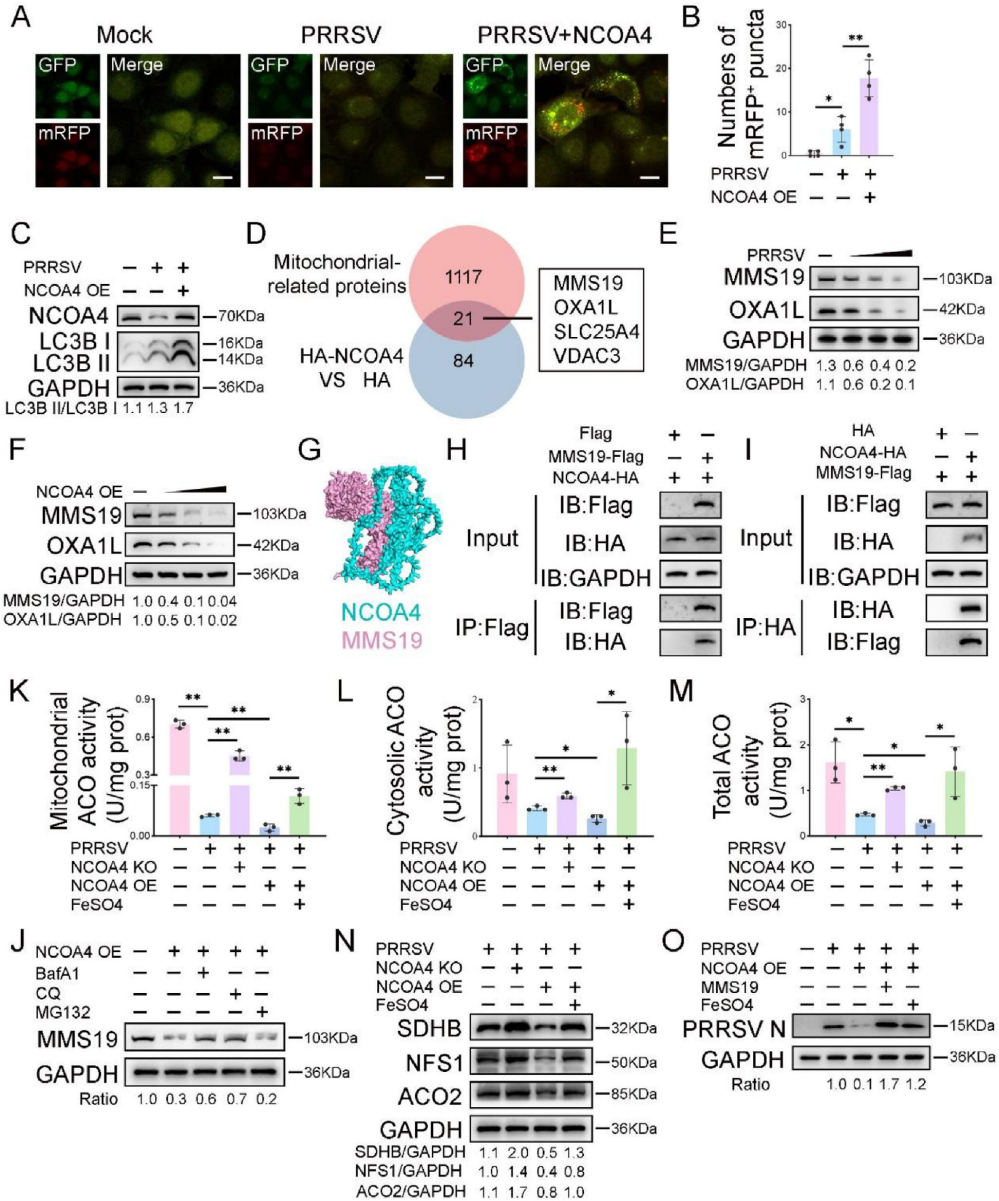
NCOA4‐mediated ferritinophagy suppresses PRRSV replication by impairing iron‐sulfur clusters biogenesis. (A) MARC‐145 cells stably expressing the LC3‑GFP‑RFP reporter were infected with PRRSV with or without NCOA4 overexpression. Cells were harvested 24 h post‐infection and analyzed by confocal microscopy. (B) The number of mRFP‑positive (mRFP^+^) puncta per cell was quantified to assess autophagic flux. ^*^ *p* < 0.05, ^**^ *p* < 0.01, Student's *t*‐test, *n* ≥ 3. (C) MARC‐145 cells were infected with PRRSV following NCOA4 overexpression or control vector transfection, with mock serving as a negative control. Cells were collected 24 h post‐infection, and the expression of LC3B II was analyzed by immunoblotting. (Original blot images in Figure S9 3a). (D) A Venn diagram illustrating potential interacting proteins identified through co‐immunoprecipitation (Co‐IP) and mass spectrometry (LC‐MS) analysis of NCOA4. (E) MARC‐145 cells were infected with PRRSV at various MOIs (0.5, 1, and 5MOI), and cells were collected 24 h post‐infection. Immunoblotting analysis was conducted to evaluate the expression levels of MMS19 and OXAIL proteins. (Original blot images in Figure S9 3b). (F) A NCOA4 recombinant plasmid was transfected into MARC‐145 cells in a concentration gradient (50, 75, and 100µg), followed by Immunoblotting analysis to assess MMS19 and OXAIL protein levels. (Original blot images in Figure S9 3c). (G) 3D structural representation illustrating the interaction between NCOA4 (blue) and MMS19 (pink). (H) (I) HEK‐293T cells were transfected with MMS19‐Flag and NCOA4‐HA along with their respective control vectors, and co‐immunoprecipitation was performed 36 h later. (Original blot images in Figure S9 3d‐3e). (J) Immunoblotting analysis of MMS19 protein expression. (Original blot images in Figure S9 3f). (K‐M) MARC‐145 cells with NCOA4 KO, NCOA4 OE, and WT were infected with PRRSV, and FeSO_4_ was added 12 h before sample collection. Mitochondrial ACO (K), cytoplasmic ACO (L), and total ACO activity (M) were assessed using an ACO detection kit. ^*^ *p* < 0.05, ^**^ *p* < 0.01, Student's *t*‐test, *n* ≥ 3. (N) (O) Immunoblotting analysis of SDHB, NFS1, ACO2 and PRRSV N protein expression. (Original blot images in Figure S9 3g‐3h).

To investigate the mechanism by which NCOA4 affects mitochondria, a stable MARC‐145 cell line that expresses LC3‐GFP‐RFP was generated. Both flow cytometry and immunofluorescence microscopy revealed that NCOA4 overexpression increased red fluorescence (indicative of autolysosomes) and the number of LC3B puncta upon PRRSV infection (Figure 3A,B). Immunoblotting indicated that both PRRSV infection and NCOA4 overexpression increased LC3‐II formation (Figure 3C; Figure S3A), while PRRSV infection downregulated FTH1 expression (Figure 1A). This suggests that PRRSV infection promotes ferritinophagy, which is exacerbated by NCOA4 overexpression. Then LC‐MS sequencing was performed on cells with or without NCOA4 overexpression using co‐immunoprecipitation (Co‐IP), revealing 28 proteins that specifically interacted with NCOA4, including 7 related to mitochondria (Figure 3D; Table S1). Among these, 4 were mitochondrial membrane proteins crucial for mitochondrial function. Immunoblotting showed that PRRSV infection downregulated MMS19 and OXA1L expression, while SLC25A4 and VDAC3 levels remained unchanged (Figure 3E; Figure S3B,D). Similarly, NCOA4 overexpression reduced MMS19 and OXA1L levels in a dose‐dependent manner, without affecting the other two proteins (Figure 3F; Figure S3C,E). This specific downregulation of MMS19 was conserved in PAM and iPAM (PAM cell lines) (Figure S3I–L). Furthermore, the findings from Co‐IP and immunoblotting assays demonstrated that NCOA4 interacts with MMS19 and mediates the autophagic degradation of MMS19 expression. (Figure 3G–J; Figure S3F).

As MMS19 is an important component protein of cellular iron‐sulfur clusters and plays a key role in maintaining mitochondrial oxidative function, it is hypothesised that NCOA4‐mediated ferritinophagy affects mitochondrial homeostasis and metabolism by autophagic degradation of MMS19, leading to damage to iron‐sulfur clusters. To investigate this hypothesis, NCOA4 expression levels were modulated in MARC‐145 cells via overexpression and knockout approaches. Additionally, treatments with Mito‐Tempo, Bafilomycin A1 (BafA1), and FeSO4 were employed to evaluate the impact of NCOA4 on iron‐sulfur clusters. UV–vis spectral analysis showed that NCOA4 overexpression decreased absorption within the 320–420 nm range (characteristic of Fe‐S clusters), whereas NCOA4 knockout increased it (Figure S3M,N). Consistently, the activity of the Fe‐S clusters‐dependent enzyme aconitase was significantly diminished in whole‐cell, cytosolic, and mitochondrial fractions upon NCOA4 overexpression, and elevated upon its knockout. FeSO_4_ supplementation rescued the aconitase activity deficit caused by NCOA4 overexpression (Figure 3K–M). We further found that NCOA4 overexpression suppressed the expression of the essential Fe‐S clusters scaffold protein NFS1 (at both mRNA and protein levels) and of the Fe‐S proteins SDHB and ACO2. These suppressive effects were partially reversed by FeSO_4_ treatment (Figure 3N; Figure S3G,H). Importantly, both MMS19 overexpression and FeSO_4_ supplementation reversed the inhibition of PRRSV replication imposed by NCOA4 (Figure 3O; Figure S3O). These results indicate that NCOA4‐mediated ferritinophagy directly damages iron‐sulfur clusters through the autophagic degradation of MMS19, leading to abnormal iron metabolism, thereby inhibiting PRRSV replication.

### 2.4 NCOA4‐Mediated Ferritinophagy Influences Lipid Droplet Formation via the Iron‐Lipid Metabolism Axis

**FIGURE 4:**
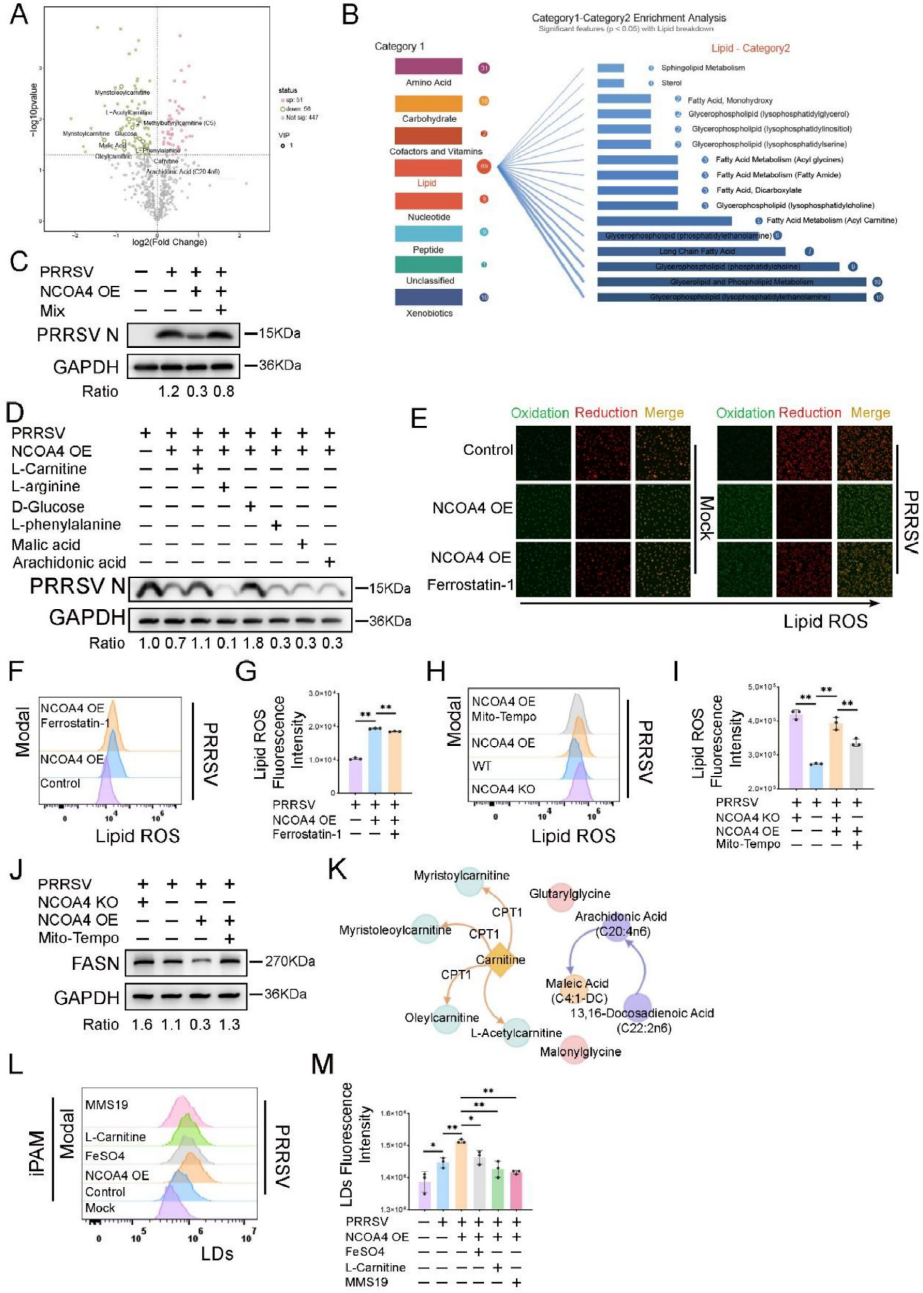
NCOA4 promotes lipid droplet formation by modulating lipid metabolism. (A) Volcano plot and (B) Metabolite category enrichment analysis of differentially expressed metabolites. (C,D) Immunoblotting analysis of PRRSV N protein expression. (E) Observation of lipid ROS fluorescence signals using confocal microscopy. (Original blot images in Figure S9 4a‐4b). (F,H) Flow cytometry analysis of lipid ROS levels, with quantitative measurement based on average fluorescence intensity (G,I), ^**^ *p* < 0.01, one‐way ANOVA, *n* ≥ 3. (J) Immunoblotting analysis of FASN protein expression. (Original blot images in Figure S9 4c). (K) Correlation network analysis of downregulated DEMs identified in (A). (L) Flow cytometry analysis of LDs levels in iPAM cells, with quantitative measurement based on average fluorescence intensity (M) ^**^ *p* < 0.01, Student's *t*‐test, *n* ≥ 3.

Alterations in mitochondrial and iron‐sulfur clusters frequently coincide with metabolic disruptions. Consistent with the sample preparation protocol used for transcriptome sequencing, metabolome sequencing identified a total of 149 differential metabolites (DEMs), with 51 upregulated and 98 downregulated (VIP > 1 and *p* < 0.05) (Figure 4A; Table S2). The majority category of DEMs were linked to lipid, specifically fatty acid metabolism (Figure 4B; Figure S4A). Integrated transcriptomic‐metabolomic analysis highlighted six downregulated small molecules linked to mitochondrial function, including L‐Carnitine, L‐arginine, D‐Glucose, L‐phenylalanine, Malic acid, and Arachidonic acid (Figure S4B). Supplementation experiments revealed that L‐Carnitine and D‐glucose most effectively reversed the NCOA4‐mediated suppression of PRRSV N protein, without cytotoxicity (Figure 4C,D; Figure S4C–E). KEGG pathway analysis also indicated that the DEMs were highly enriched in lipid metabolism pathways (Figure S4F), suggesting that NCOA4‐mediated ferritinophagy inhibits PRRSV replication by affecting the iron‐lipid metabolism axis. To test this, the levels of lipid peroxidation within the cells were measured. Flow cytometry and immunofluorescence microscopy showed that NCOA4 overexpression significantly increased lipid peroxidation in PRRSV‐infected cells, an effect suppressed by Mito‐Tempo (Figure 4E–I). Given that lipid metabolism is tightly regulated by key enzymes such as fatty acid synthase (FASN), we examined its expression. Immunoblotting showed that NCOA4 overexpression downregulated FASN protein levels, while NCOA4 knockout upregulated them. Mito‐Tempo treatment rescued the FASN downregulation induced by NCOA4 (Figure 4J; Figure S4G). Further analysis of DEMs revealed significant changes in the fatty acid metabolism pathway, particularly related to Carnitine (Figure 4K; Figure S4H). Since L‐Carnitine is essential for transporting long‐chain fatty acids into mitochondria for β‐oxidation [23], we asked whether L‐carnitine deficiency contributed to the observed metabolic phenotype. Flow cytometry showed that NCOA4 overexpression significantly increased lipid droplet formation, while L‐Carnitine treatment inhibited this effect both in iPAM and MARC‐145 cells (Figure 4L,M; Figure S4I,J), suggesting that L‐Carnitine can counteract NCOA4's promotion of lipid droplet formation. The results also indicate that MMS19 and FeSO_4_ have the same effect on LDs as L‐Carnitine in this process (Figure 4L,M). These findings suggesting that NCOA4‐mediated ferritinophagy inhibits PRRSV replication by affecting a coordinated iron–lipid metabolism axis. Specifically, it influences this axis to promote lipid droplet formation, a process linked to intracellular carnitine levels, thereby demonstrating that iron homeostasis directly regulates lipid metabolic reprogramming.

### 2.5 The Function of Carnitine in the Formation of Lipid Droplets

**FIGURE 5:**
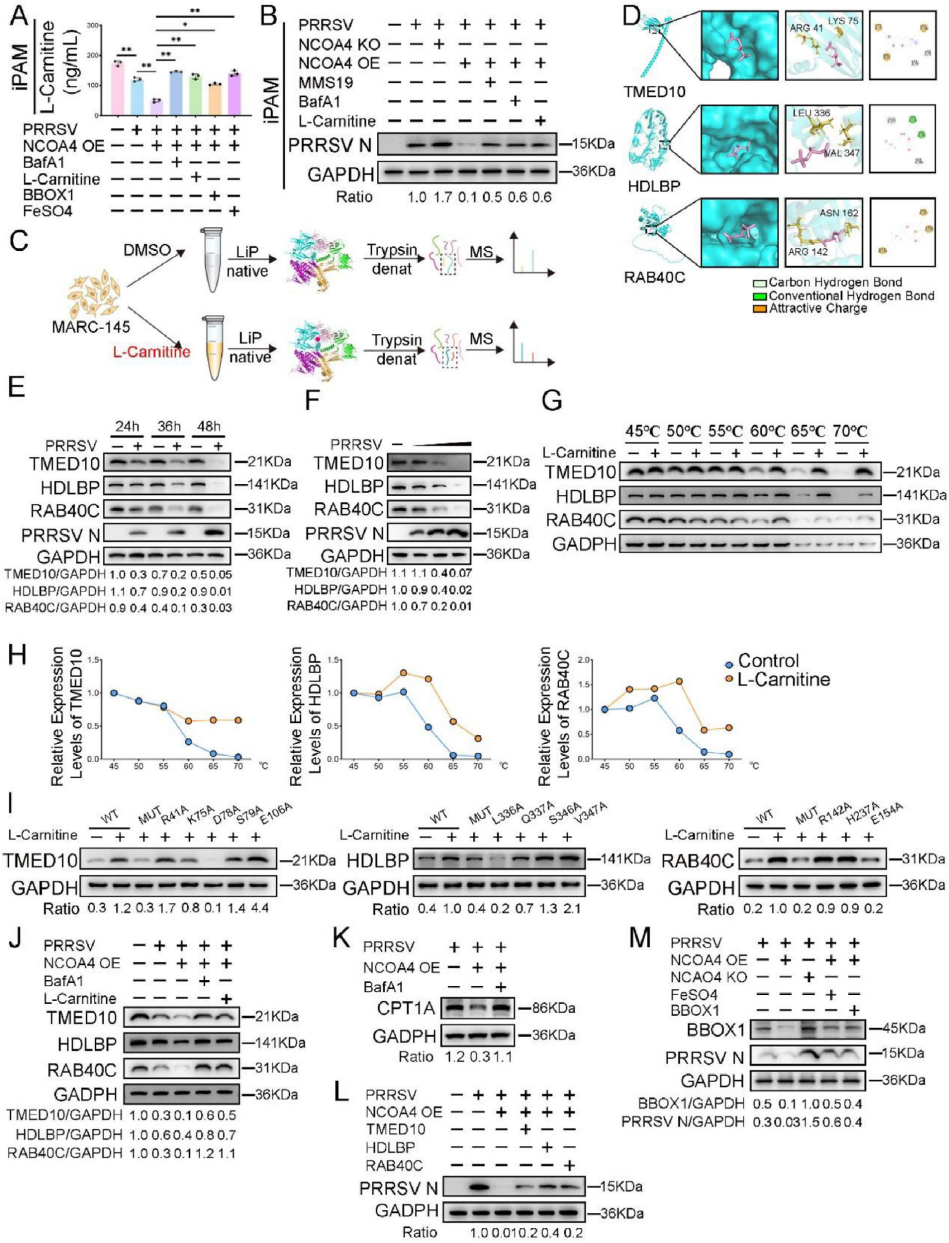
Interaction mechanism of L‐Carnitine and Lipid Droplets (A) Intracellular L‑Carnitine levels in iPAM cells were measured using an L‐Carnitine detection kit, ^**^ *p* < 0.01, one‐way ANOVA, *n* = 3. (B) Immunoblotting analysis was used to detect the expression of BBOX1 protein in iPAM cells. (Original blot images in Figure S9 5a). (C) Schematic workflow of the LiP‐SMap experiment used to identify L‑Carnitine‑binding proteins. (D) Optimal molecular docking models of TMED10, HDLBP, RAB40C, and L‐Carnitine. The left column shows a schematic diagram of the overall structure, the second column uses a surface filling model, where cyan represents the surface structure of the protein and pink indicates L‐Carnitine; the third column displays the amino acid residues in a 3D structure; the far‐right column presents a 2D representation showing key amino acid residues and the interaction network between the residues. (E,F) MARC‐145 cells infected with PRRSV were collected at different times (E) or different concentration gradients (0.5MOI, 1MOI, and 5MOI) (F). Immunoblotting analysis was used to detect the expression of TMED10, HDLBP, and RAB40C proteins. (Original blot images in Figure S9 5b‐5c). (G,H) MARC‐145 cell lysates were treated with L‐Carnitine (20µm) or DMSO for 1 h, followed by heating at different temperatures for 3 min. (G) Immunoblotting analysis was used to detect the expression of TMED10, HDLBP, and RAB40C proteins, and (H) quantitative analysis of the protective effect of L‐Carnitine on TMED10, HDLBP, and RAB40C proteins. (Original blot images in Figure S9 5d). (I) Wild‐type and mutant plasmids were transfected, and immunoblotting analysis was used to detect protein expression, respectively. (Original blot images in Figure S9 5e). (J,K) Immunoblotting analysis was used to detect the expression of TMED10, HDLBP, RAB40C (J) and CPT1A (K) proteins. (Original blot images in Figure S9 5f‐5g). (L) Overexpress TMED10, HDLBP, and RAB40C respectively while overexpressing NCOA4 in MARC‐145 cells, followed by infection with PRRSV. Immunoblotting analysis was used to detect the expression of PRRSV N protein. (Original blot images in Figure S9 5h). (M) Immunoblotting analysis was used to detect the expression of BBOX1 protein. (Original blot images in Figure S9 5i).

To further investigate how Carnitine affects lipid droplet synthesis during infection, intracellular L‐Carnitine levels were measured in PRRSV‐infected cells. ELISA confirmed that NCOA4 overexpression significantly reduced intracellular L‐Carnitine levels in PRRSV‐infected cells, which was restored by L‐Carnitine supplementation, FeSO_4_ treatment, or overexpression of γ‐butyrobetaine hydroxylase 1 (BBOX1), a key enzyme in carnitine biosynthesis (Figure 5A; Figure S5B). Restoring L‐Carnitine levels correlated with increased PRRSV N protein (Figure 5B). Subsequently, potential targets of L‐Carnitine were identified using LiP‐Smap assay (Figure 5C). LC‐MS results revealed that several proteins interacting with L‐Carnitine, including TMED10, HDLBP, and RAB40C, are related to lipid droplet synthesis (Figure S5A, Table S3). Molecular docking assay showed that L‐Carnitine could directly interact with these three proteins (Figure 5D). Furthermore, PRRSV infection resulted in a time‐ and dose‐dependent reduction in the protein expression levels of TMED10, HDLBP, and RAB40C (Figure 5E,F; Figure S5D,E). To explore the protective role of L‐Carnitine on TMED10, HDLBP, and RAB40C, a Cellular Thermal Shift Assay (CETSA) experiment was performed for confirmation. The immunoblotting results indicated that L‐Carnitine decreased the degradation of TMED10, HDLBP, and RAB40C proteins caused by temperature changes (Figure 5G,H). This indicates that L‐Carnitine provides stabilizing effects for all three proteins. To confirm this conclusion, molecular docking results were combined, and mutations were introduced at the amino acid interaction sites. Subsequently, wild‐type or mutated plasmids were transfected. The immunoblotting results showed that after mutation, the protective effect of L‐Carnitine on TMED10 (Asp78), HDLBP (Leu336), and RAB40C (Glu154) were lost, indicating that these sites are specifically involved in the interaction between L‐Carnitine and TMED10, HDLBP, and RAB40C (Figure 5I; Figure S5F). Furthermore, both the autophagy inhibitor BafA1 and L‐Carnitine supplementation alleviated the NCOA4‐mediated suppression of these proteins during infection (Figure 5J; Figure S5G). The results also showed that overexpression of each of these three proteins significantly restored the levels of the PRRSV N protein (Figure 5L; Figure S5I). Mechanistically, NCOA4 overexpression downregulated carnitine palmitoyltransferase I (CPT1), a critical enzyme for mitochondrial fatty acid import [24, 25], and the biosynthetic enzyme BBOX1 (Figure 5K,M; Figure S5H,J). In summary, these data delineate a mechanism whereby NCOA4‐mediated ferritinophagy depletes L‐Carnitine, leading to the destabilization of key LD regulators (TMED10, HDLBP, RAB40C). Crucially, we demonstrate that carnitine directly stabilizes these lipid droplet‐associated proteins. This cascade promotes LDs accumulation and culminates in the inhibition of PRRSV replication.

### 2.6 NCOA4‐Mediated ferritinophagy Inhibits Iron‐Dependent Viral Replication via the Iron‐Lipid Metabolism Axis

**FIGURE 6:**
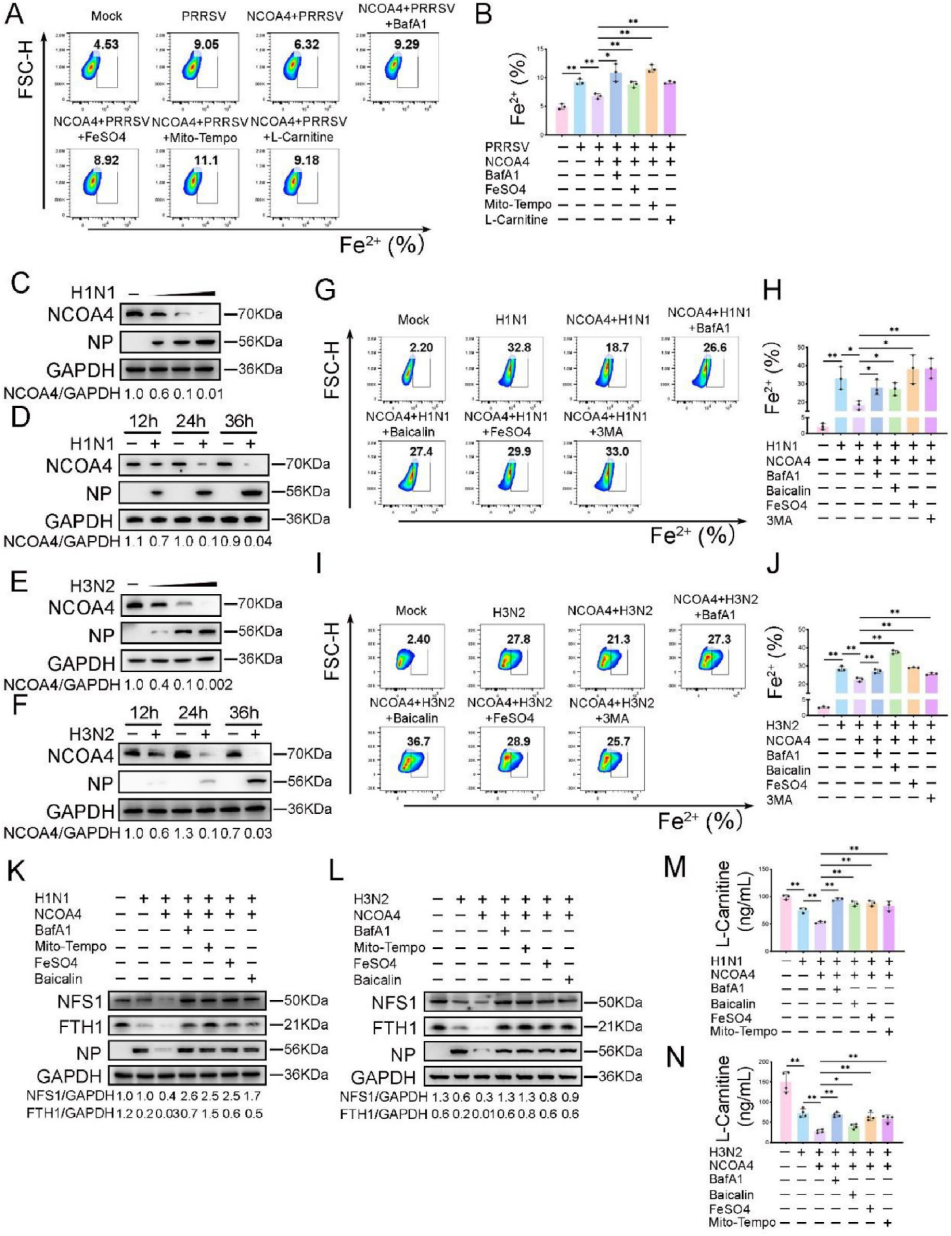
NCOA4‐mediated iron‐lipid metabolism axis broadly inhibits iron‐dependent viral replication. (A) Flow cytometry was used to analyze intracellular Fe^2+^ levels during PRRSV infection; (B) Quantitative assessment of Fe^2+^ levels was conducted based on the percentage of positive cells. ^*^ *p* < 0.05, ^**^ *p* < 0.01, Student's *t*‐test, *n* ≥ 3. (C–F) Expression of NCOA4 and influenza virus nucleoprotein (NP) in infected MDCK cells. (C,D) Cells were infected with H1N1 at the indicated MOIs (0.5, 1, and 5MOI) (C) or with an MOI of 1 for the indicated times (D). (E,F) Parallel experiments performed with H3N2 infection. Cell lysates were harvested at 36 h (C,E) or at the indicated times (D,F) post‐infection and analyzed the protein expression of NCOA4 and NP. (Original blot images in Figure S9 6a‐6d). (G–J) Intracellular Fe^2^^+^ levels in influenza virus‑infected MDCK cells. (G,H) Cells infected with H1N1. (I,J) Cells infected with H3N2. (G,I) Representative flow cytometry histograms. (H,J) A quantitative analysis of Fe^2+^ levels was conducted based on the percentage of positive cells. ^*^ *p* < 0.05, ^**^ *p* < 0.01, Student's *t*‐test, *n* ≥ 3. (K,L) MDCK cells were infected with H1N1 (K) or H3N2 (L). Immunoblotting was used to analyze the expression of NFS1, FTH1, and NP proteins. (Original blot images in Figure S9 6e‐6f). (M,N) Intracellular L‑Carnitine levels in MDCK cells infected with H1N1 (M) or H3N2 (N). ^*^ *p* < 0.05, ^**^ *p* < 0.01, Student's *t*‐test, *n* ≥ 3.

Our data suggest that NCOA4 disrupts Fe‐S clusters biogenesis, depletes the Fe^2+^ pool, and thereby inhibits the Fe^2+^‐dependent enzyme BBOX1, leading to L‐Carnitine deficiency. To directly test the central role of Fe^2+^ depletion in this axis, we measured intracellular Fe^2+^ levels. Flow cytometry confirmed that NCOA4 overexpression reduced Fe^2+^ levels in PRRSV‐infected cells, which was restored by treatment with BafA1, FeSO_4_, Mito‐Tempo, or L‐Carnitine (Figure 6A,B). We next asked whether this antiviral mechanism extends to other iron‐dependent viruses. Infection with influenza A viruses (H1N1 and H3N2) downregulated NCOA4 protein expression in a time‐ and dose‐dependent manner (Figure 6C–F; Figure S6A–D), mirroring our observations with PRRSV. Importantly, NCOA4 overexpression similarly reduced Fe^2+^ levels in H1N1‐ or H3N2‐infected cells. This reduction was reversed by BafA1, FeSO_4_, the CPT1 activator baicalin, or the autophagy inhibitor 3‐MA (Figure 6G–J). Consistent with disrupted Fe‐S clusters biogenesis, NCOA4 also downregulated the Fe‐S clusters scaffold protein NFS1 during influenza infection, an effect alleviated by the same treatments (Figure 6K,L; Figure S6E,F). Finally, ELISA showed that influenza infection decreased L‐Carnitine levels, which was exacerbated by NCOA4 overexpression and rescued by BafA1, baicalin, FeSO_4_, or Mito‐Tempo (Figure 6M,N). These results demonstrate that the NCOA4/ferritinophagy/Fe‐S/Fe^2+^/carnitine axis constitutes a conserved host defense mechanism against diverse iron‐dependent viruses, including PRRSV and influenza A virus.

### 2.7 PRRSV NSP5 Induces Autophagic Degradation of NCOA4

**FIGURE 7:**
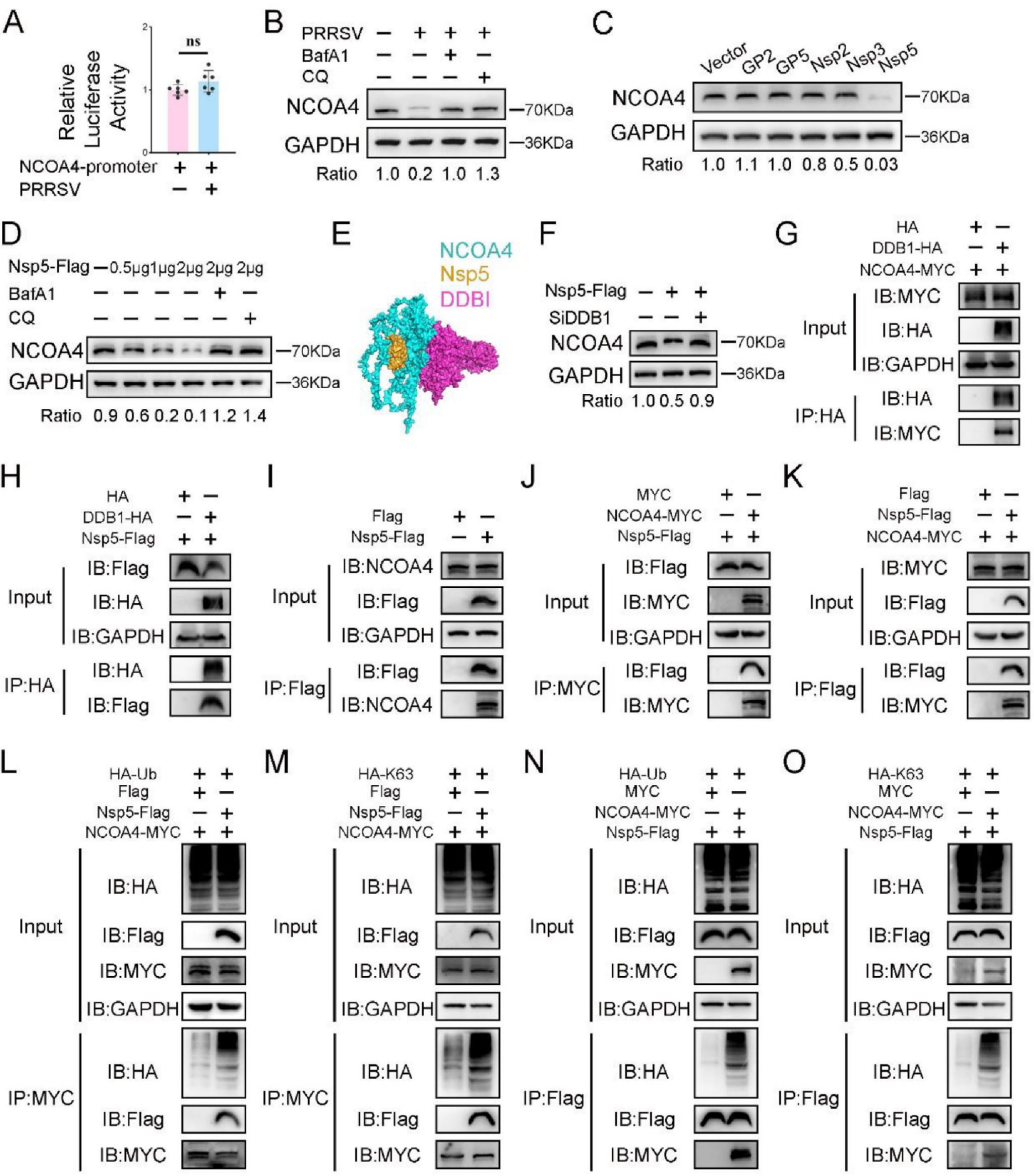
PRRSV Nsp5 downregulates NCOA4 via ferritinophagy. (A) NCOA4 promoter activity was assessed by a dual‑luciferase reporter assay. MARC‐145 cells were co‐transfected with the reporter plasmid and a reference plasmid, followed by PRRSV infection. ns indicates not significant, Student's *t*‐test, *n* ≥ 3. (B) MARC‐145 cells were infected with PRRSV (MOI = 1), and inhibitors BafA1 and CQ were added 12 h prior to sample collection. Immunoblotting analysis was used to detect NCOA4 protein expression. (Original blot images in Figure S9 7a). (C,D) Screening for PRRSV proteins that regulate NCOA4. (C) HEK‑293T cells were transfected with plasmids encoding empty vector, GP2, GP5, Nsp2, Nsp3, or Nsp5. (D) HEK‑293T cells were transfected with increasing amounts of an Nsp5‑Flag plasmid or the Flag‑empty vector control. Cells were harvested 36 h post‑transfection, and immunoblotting analysis was used to detect NCOA4 protein expression. (Original blot images in Figure S9 7b‐7c). (E) 3D structural representation illustrating the interaction among Nsp5 (yellow), NCOA4 (blue) and DDB1 (pink). (F) HEK‑293T cells were transfected to overexpress Nsp5, along with siRNA targeting DDB1 (siDDB1). Immunoblotting analysis was used to detect NCOA4 protein expression. (Original blot images in Figure S9 7d). (G–K) Co‑IP assays to examine interactions among Nsp5, DDB1, and NCOA4. (G,H) HEK‐293T cells were transfected with NCOA4‐MYC or Nsp5‐Flag along with DDB1‐HA or HA empty vector. (I) HEK‐293T cells were transfected with Flag empty vector and Nsp5‐Flag recombinant plasmids. (J,K) HEK‐293T cells were transfected with NCOA4‐MYC and Nsp5‐Flag or the corresponding empty vector. Cell lysates were collected 36 h post‐transfection, followed by affinity separation, and co‐immunoprecipitation analysis with specific antibodies. (Original blot images in Figure S9 7e‐7i). (L–O) HEK‐293T cells were co‐transfected with Flag empty vector or Nsp5‐Flag and NCOA4‐MYC along with HA‐Ub or HA‐K63, followed by affinity separation using anti‐MYC or anti‐Flag beads and co‐immunoprecipitation analysis with specific antibodies. (Original blot images in Figure S9 7j‐7m).

The previous results demonstrated that PRRSV infection downregulates NCOA4 expression. To determine if PRRSV affects NCOA4 transcription, dual‐luciferase assays showed no significant difference in promoter activity between PRRSV‐infected and control groups (Figure 7A), indicating that PRRSV does not regulate *NCOA4* transcriptionally. Whether PRRSV induces NCOA4 degradation through autophagy was investigated using inhibitors BafA1 and Chloroquine (CQ). Immunoblotting revealed that these treatments restored NCOA4 expression levels reduced by PRRSV infection (Figure 7B; Figure S7A), suggesting that PRRSV degrades NCOA4 via autophagy rather than transcriptional regulation. To identify the viral factor responsible, we screened PRRSV proteins reported to modulate autophagy. Overexpression in HEK‐293T cells showed that only Nsp5 downregulated NCOA4 in a dose‐dependent manner (Figure 7C,D; Figure S7B,C). To investigate the mechanism by which Nsp5 targets NCOA4, we initially analyzed mass spectrometry data obtained from NCOA4 co‐immunoprecipitation experiments. This analysis revealed the presence of DDB1, a fundamental constituent of the CRL4 E3 ubiquitin ligase complex. Computational predictions further suggested possible interactions among Nsp5, DDB1, and NCOA4 (Figure 7E). Knockdown of DDB1 by siRNA attenuated the Nsp5‐mediated suppression of NCOA4 (Figure 7F; Figure S7D). Reciprocally, co‐immunoprecipitation confirmed interactions among Nsp5, DDB1, and NCOA4 (Figure 7G–K). Given the link between ubiquitination and autophagic degradation, we examined NCOA4 ubiquitination. Nsp5 expression increased total ubiquitination of NCOA4 and specifically enriched for K63‐linked polyubiquitin chains, a canonical signal for autophagic clearance (Figure 7L–O). A similar interaction pattern was observed between Nsp5 and MMS19 (Figure S7E–K). The above results demonstrate that PRRSV Nsp5 promotes the autophagic degradation of NCOA4 by recruiting the E3 ligase component DDB1 to mediate K63‐linked ubiquitination of NCOA4.

## 3 Discussion

This study identifies NCOA4‐mediated ferritinophagy as a pivotal regulator of iron‐sulfur clusters homeostasis and lipid metabolic reprogramming, thereby establishing a novel antiviral mechanism orchestrated by the “iron‐lipid metabolism axis”—a conceptual framework in which cellular iron homeostasis, governed by ferritinophagy, directly dictates lipid metabolic reprogramming to determine host permissiveness to viral infection. We further reveal how PRRSV counteracts this axis via Nsp5‐mediated degradation of NCOA4, highlighting an evolutionary conflict centered on the control of ferritinophagy. Supporting this antagonism, transcriptomic reanalysis of PRRSV‐resistant pig breeds (Tibetan/Tongcheng vs susceptible Large White; PiggTEx database) shows constitutively elevated NCOA4 expression in the lungs, spleen, and alveolar macrophages (PAMs) [26, 27, 28] (Figure S8A). Furthermore, within susceptible breeds, NCOA4 levels inversely correlate with viral titers during infection [29] (Figure S8B), indicating that PRRSV actively suppresses NCOA4 to evade host restriction. Thus, NCOA4‐mediated ferritinophagy emerges as a critical determinant in host‐virus evolutionary dynamics, where metabolic reprogramming directly dictates infection outcomes.

NCOA4‐mediated ferritinophagy coordinates intracellular iron flux and plays a critical role in regulating viral infections [30]. While previous studies have demonstrated its antiviral effects against Human Cytomegalovirus (HCMV) and human Parainfluenza Virus 2 (hPIV2) [31, 32], PRRSV subverts this pathway by downregulating NCOA4 and FTH1 and accumulating LC3‐II, indicating an enhancement of ferritinophagy. Our work establishes NCOA4 as a key host restriction factor against PRRSV, extending its role beyond iron mobilization. Importantly, we identify MMS19 as a novel NCOA4 interactor and demonstrate that its autophagic degradation is the mechanism by which ferritinophagy disrupts Fe‐S clusters—essential cofactors for viral enzymes (e.g., SARS‐CoV‐2 RdRp) and metabolic stability [2, 33]. This NCOA4‐driven process reduces Fe‐S clusters abundance, downregulates the scaffold proteins NFS1/SDHB/ACO2, and impairs aconitase activity, directly compromising mitochondrial function. Mechanistically, our study provides the first evidence that: (i) ferritinophagy triggers mitochondrial metabolic remodeling via Fe‐S clusters disruption; (ii) viral immune evasion targets this pathway by degrading its key regulator (NCOA4) through a precise mechanism involving viral protein recruitment of DDB1; and (iii) this antiviral axis functions independently of canonical immune signaling. Notably, while NCOA4‐mediated ferritinophagy has been characterized primarily in cancer contexts where it supports tumor growth by supplying iron for proliferation [34], our work establishes its distinct and protective role during viral infection. In cancer, defects in Fe‐S clusters biosynthesis are often associated with metabolic reprogramming that promotes anabolic processes and chemoresistance [35, 36]; however, during viral infection, we demonstrate that NCOA4 binding to MMS19 induces its autophagic degradation, leading to Fe‐S clusters impairment and consequent suppression of viral replication. This highlights the dual role of Fe‐S clusters biogenesis, wherein its disruption is pathogenic in cancer but protective in antiviral defense, with our study establishing this mechanism specifically within the context of host‐virus interaction.

Fe‐S clusters critically regulate lipid metabolism by modulating mitochondrial function and the activity of metabolic enzymes [37]. Imbalanced Fe‐S homeostasis impairs fatty acid β‐oxidation and electron transport, leading to lipid accumulation. For example, defective Fe‐S assembly reduces complex I activity and ATP production, thereby promoting lipogenesis [38]. Key Fe‐S‐dependent enzymes, such as aconitase (ACO2), regulate fatty acid metabolism across various systems [39, 40]. Moreover, Fe‐S deficiency elevates citrate levels, which drives fatty acid synthesis and lipid droplet (LD) formation [41]. Our metabolomic analyses reveal that NCOA4 overexpression disrupts this regulatory axis: reduced Fe^2^^+^ availability (from Fe‐S collapse and suppressed STEAP3/GSH [37, 42, 43, 44]) inhibits BBOX1, an iron‐dependent carnitine synthase [45]. This inhibition depletes L‐Carnitine, impairing mitochondrial fatty acid transport and β‐oxidation [46, 47], thereby redirecting metabolism toward LD accumulation. We define a novel mechanistic cascade linking ferritinophagy to lipid reprogramming: NCOA4 induces MMS19 degradation, causing Fe‐S clusters disruption and decreased Fe^2^^+^ levels, this suppresses BBOX1 activity and L‐Carnitine synthesis, ultimately promoting LD accumulation. Importantly, L‐Carnitine supplementation rescues NCOA4‐induced LD accumulation and antiviral effects, directly demonstrating that Fe‐S‐driven mitochondrial remodeling reprograms lipid metabolism via carnitine suppression to achieve antiviral defense.

Lipid droplets (LDs) play a critical role in viral replication, with TMED10, HDLBP (vigilin), and RAB40C identified as key regulators of LD dynamics. TMED10 suppression increases LD size and number [48], HDLBP prevents pathological cholesterol accumulation [49], and the RAB40C GTPase promotes lipophagy—where its overexpression enhances LD degradation, while knockdown leads to accumulation [50]. Notably, we discovered that L‐Carnitine stabilizes these LD regulators to maintain lipid homeostasis. Using integrated LiP‐SMap, molecular docking, and CETSA approaches, we mapped L‐Carnitine binding sites on TMED10, HDLBP, and RAB40C, with site‐directed mutagenesis confirming their functional importance. CETSA further demonstrated enhanced thermal stability of these proteins upon L‐Carnitine binding. Crucially, PRRSV infection downregulated TMED10, HDLBP, and RAB40C expression, indicating viral disruption of LD homeostasis to facilitate replication, consistent with known viral exploitation of lipid metabolism [51, 52]. We propose that L‐Carnitine prevents virus‐induced degradation of these proteins through structural stabilization, thereby preserving LD integrity. This carnitine‐mediated protection of LD regulatory machinery represents a novel host‐defense mechanism that therapeutically targets viral lipid dependency. Our findings extend the functional repertoire of lipid metabolism in host defense and underscore its dual nature: while cancer cells hijack this pathway to support proliferation [53], the host employs it for antiviral immunity, with L‐Carnitine serving as a key metabolic node in this protective response.

The core of this study is to validate the NCOA4‐mediated ferritinophagy/iron‐sulfur protein/iron/carnitine/lipid meta‐bolism axis as a novel antiviral mechanism. We demonstrate that knocking out NCOA4 enhances viral replication, whereas its overexpression inhibits the replication of various RNA viruses, such as multiple PRRSV strains (classical strain, highly pathogenic strain, NADC30 strain) and influenza A viruses (H1N1, H3N2). Additionally, previous studies have shown that the replication of certain DNA viruses is also closely related to ferritinophagy; for example, PRV infection can induce NCOA4‐mediated ferritinophagy to promote its own replication [54], while ferritinophagy can significantly suppress HCMV replication [31]. Therefore, this antiviral mechanism exhibits the potential to have broad‐spectrum activity. In this study, viral infections actively downregulate NCOA4, FTH1, and L‐Carnitine to subvert lipid metabolic reprogramming, confirming the “iron‐lipid metabolism axis” as a universal defense strategy against iron‐dependent viruses. Although PRRSV Nsp5 (an ER transmembrane protein) was previously known to enhance replication via autophagy induction [55, 56], we reveal its role as a master immune evader. Immunoblotting and luciferase assays with autophagy inhibitors demonstrate that Nsp5 suppresses NCOA4 through autophagic degradation rather than transcriptional repression. Critically, co‐immunoprecipitation reveals that Nsp5 interacts with NCOA4 and recruits DDB1 to catalyze its K63‐linked polyubiquitination, a canonical signal for autophagic degradation [57, 58]. This defines a precise countermeasure: viral hijacking of the host ubiquitin‐autophagy system to dismantle the metabolic defense axis.

**FIGURE 8:**
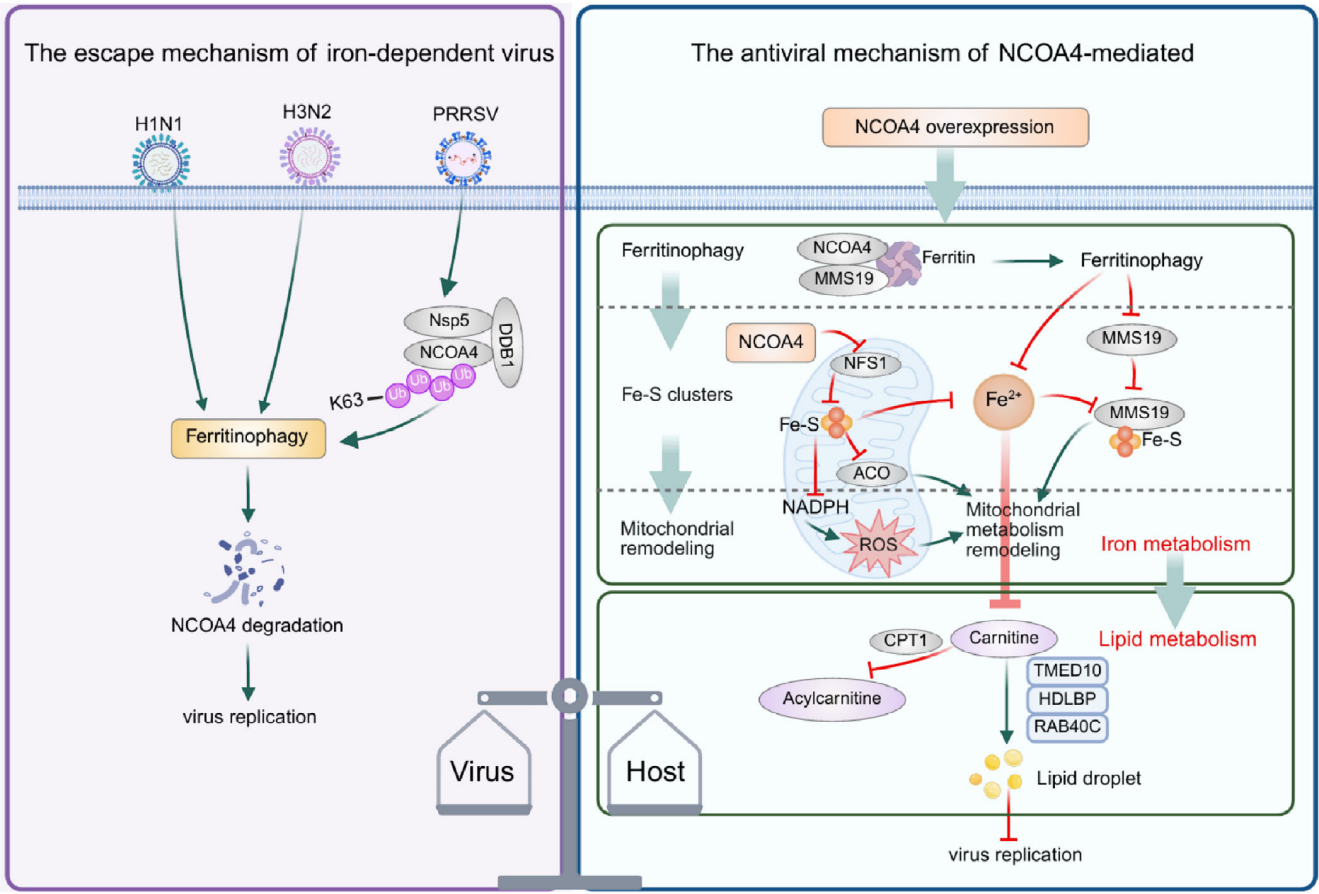
Schematic model of viral inhibition via NCOA4‑dependent ferritinophagy (Created with BioRender.com).

In summary, this study establishes the “iron‐lipid metabolism axis” (Figure 8) as an innovative paradigm for broad‐spectrum antiviral defense. This axis is built upon three interlinked mechanisms: NCOA4‐mediated degradation of MMS19 to disrupt Fe‐S clusters, carnitine‐mediated stabilization of lipid droplet proteins, and viral antagonism via Nsp5‐DDB1‐mediated degradation of NCOA4. Therapeutic strategies targeting this axis—including activators of ferritinophagy/carnitine pathways or inhibitors of Nsp5‐mediated ubiquitination—hold significant promise. Future investigations should prioritize: (1) elucidating the mechanisms underlying BBOX1 – Fe^2^^+^ dependence, including direct measurement of BBOX1 enzymatic activity under varying iron conditions; (2) defining the structural basis and therapeutic potential of carnitine‐mediated protein stabilization; (3) validating the in vivo efficacy of interventions targeting this axis; and (4) exploring this pathway across diverse iron‐dependent pathogens.

## 4 Materials and Methods

### 4.1 Cells and Viruses

The MARC‐145, MDCK, and HEK‐293T cell lines were cultured at the Key Laboratory of Animal Immunology, Henan Academy of Agricultural Sciences. The PAM (porcine alveolar macrophage) and iPAM (immortalized PAM) cell lines were generously provided by Huazhong Agricultural University. All cell lines were cultured in DMEM (12100, Beijing Solarbio Science & Technology Co., Ltd., China) supplemented with 10% fetal bovine serum (FS301‐02, TransGen Biotech, China). The Porcine Reproductive and Respiratory Syndrome Virus (PRRSV) strains HN07‐1, A/Puerto Rico/8/1934 (PR8, H1N1), and A/swine/Henan/1/2010 (H3N2) were isolated and stored in our laboratory. Additionally, the highly pathogenic PRRSV strain (HuB‐1‐China‐2021), NADC3‐like strain (HeN11001‐2023), and classical VR2332 strains (SC12069‐2023 and BJ‐04) were provided by Huazhong Agricultural University. Unless otherwise specified, the HN07‐1 strain was used in this study.

### 4.2 Antibodies

The antibodies utilized in this research included: Anti‐NCOA4 antibody (1:1000); Anti‐SDHB antibody (1:1000); Anti‐Aconitase 2 antibody (1:1000); Anti‐BBOX1 antibody [EPR11309] (ab314553, ab175225, ab129069, ab171959, Abcam, UK); Ferritin Heavy Chain Rabbit mAb (1:1000) (A19544, ABclonal, China); PRRS virus nucleocapsid protein antibody (1:1000) (GTX637650, GeneTex, USA); Rabbit monoclonal antibodies against cytochrome c (136F3) (1:1000), COX IV (3E11) (1:1000), and LC3B (E5Q2K) (1:1000) (4280T, 4850T, 83506T, Cell Signaling Technology, USA); HRP‐conjugated goat anti‐rabbit IgG (H+L) (1:5000) and goat anti‐mouse IgG (H+L) (1:5000); GAPDH polyclonal antibody (1:20000); MMS19 monoclonal antibody (1:1000); OXA1L monoclonal antibody (1:5000); SLC25A4 polyclonal antibody (1:1000); VDAC3 recombinant antibody (1:5000); NFS1 monoclonal antibody (1:5000); HDLBP polyclonal antibody (1:1000); CPT1A monoclonal antibody (1:5000); TMP21 monoclonal antibody (TMED10) (1:5000) (SA00001‐2, SA00001‐1, 10494‐1‐AP, 66049‐1‐Ig, 66128‐1‐Ig, 30631‐1‐AP, 82666‐14‐RR, 67021‐1‐Ig, 15406‐1‐AP, 66039‐3‐Ig, 67876‐1‐Ig, Proteintech, China); Anti‐FASN mouse monoclonal antibody (D190620, Sangon Biotech, China) (1:1000); Rab 40C antibody (H‐8) (1:1000) (sc‐514826, Santa Cruz Biotechnology, USA); and HRP‐conjugated mouse monoclonal antibodies against Flag Tag (1:1000), HA Tag (1:1000), and Myc Tag (1:1000) (AF2855, AF2861, AF2867, Beyotime Biotech Inc, China).

### 4.3 Plasmid Transfection

Transfection methods were selected based on the specific cell type. For MARC‐145 cells, electroporation was used, involving the transfection of 50 µg of plasmid DNA into the cells via an electroporation system. The electroporation transfection parameters are set to 850 V voltage and 950 µF capacitance. In addition, all other cell types in this study were transfected using TransIntro PL Transfection Reagent (FT301, TransGen Biotech, China).

### 4.4 SiRNA Transfection

Small interfering RNA (SiRNA) was used at a final concentration of 100nM. The siDDB1 target sequence was AACGGCUGCGUGACCGGACAC. HEK‐293T cell lines were seeded at cell culture plate. After 8 h of transfection (RNAiMAX, Thermo Fisher Scientific), the transfection mixture was replaced with fresh medium containing 10% FBS. Cells were harvested between 36 and 48 h after transfection.

### 4.5 Quantitative Real‐Time PCR

Total RNA was extracted from the cells using RNAiso Plus (9109, Takara Bio Inc., China). cDNA synthesis was performed using the PrimeScript RT Reagent Kit with gDNA Eraser (RR047A, Takara Bio Inc., China) following the manufacturer's protocol. The expression levels of target gene mRNAs were quantified using the TB Green Premix Ex Taq II (Tli RNaseH Plus) kit (RR820A, Takara Bio Inc, China), with normalization to GAPDH. The fold change in expression was calculated using the 2^−△△CT^ method. A list of primers used was provided below.

**Table:** 

Primers	Sequence (5′‐3′)
ORF7 (N)‐F	AAAACCAGTCCAGAGGCAAG
ORF7 (N)‐R	CGGATCAGACGCACAGTATG
GAPDH‐F	TGACAACAGCCTCAAGATCG
GAPDH‐R	GTCTTCTGGGTGGCAGTGAT
NFS1‐F	CACTCCCGGACACATGCTTAT
NFS1‐R	TGTCTGGGTGGTGATCAAGTG

### 4.6 Immunoblotting

Cells were lysed using RIPA lysis buffer containing 1% PMSF (P0013B, Beyotime Biotech Inc., Shanghai, China). Following denaturation, proteins were separated by SDS‐PAGE and transferred onto a 0.22 µm PVDF membrane (ISEQ00010, Millipore, Germany). The membrane was blocked at room temperature with 5% non‐fat milk for 1 h, followed by overnight incubation with the appropriate primary antibody at 4°C. After a 1 h incubation with the corresponding secondary antibody at room temperature, signals were detected using NcmECL Ultra (P10200, NCM Biotech, China).

### 4.7 Transcriptomics

MARC‐145 cells were either transfected to overexpress NCOA4 or left unmodified prior to PRRSV infection (MOI = 1). Cells were harvested 24 h post‐infection, and total RNA was extracted. After quality control, mRNA sequencing libraries were constructed and sequenced. Subsequent data analysis included differential gene expression screening and functional pathway analysis of the differentially expressed genes. Differentially expressed genes (DEGs) were identified using thresholds of fold change > *1.5* and *p* < *0.05*.

### 4.8 Metabolomics

As in the transcriptomics setup, MARC‐145 cells were first transfected to overexpress NCOA4 or left untreated as controls, then infected with PRRSV (MOI = 1). At 24 h post‐infection, cells were harvested, and metabolites were extracted. All small‐molecule metabolites in the samples were analyzed using a high‐resolution liquid chromatography‐tandem mass spectrometry platform. After peak alignment and normalization, metabolites were annotated against an in‐house library. Differential abundance and pathway enrichment analyses were performed between the NCOA4‐overexpression and control groups. Differential metabolites were identified using thresholds of VIP > 1 and *p* < *0.05*.

### 4.9 NADPH Assay

Cells were collected and treated with the Enhanced NADP+/NADPH Assay Kit with WST‐8 (S0180S, Beyotime Biotech Inc, Shanghai, China), with all cell samples were heated at 60°C to measure NADPH concentrations across different samples.

### 4.10 Transmission Electron Microscopy (TEM)

Cultured cells were washed with culture medium, fixed at room temperature with 2.5% glutaraldehyde for 5 min, and then collected by scraping. The cells were centrifuged at 2000 rpm for 2 min, followed by fixation, dehydration, and embedding in resin prior to imaging (HITACHI H‐7000FA, Tokyo, Japan).

### 4.11 Mitochondrial Isolation

Cells were harvested, and mitochondria and cytoplasmic proteins were isolated using a mitochondrial isolation kit (C3601, Beyotime Biotech Inc, China). Immunoblot analysis was subsequently performed to assess the release of mitochondrial protein cytochrome C into the cytoplasm.

### 4.12 Flow Cytometry

Following cell collection, an appropriate volume of dye was added for incubation. After thorough washing, the cells were analyzed using a flow cytometer, with data processed using FlowJo v10.8.1 software. The dyes employed in this study included MitoSOX Red, BODIPY 581/591 C11 (M36007, D3861, Thermo Fisher Scientific, USA), BODIPY 493/503 Staining Kit (C2053S, Beyotime Biotech Inc, China), and FerroOrange (Fe^2+^ indicator) (MX4559, MKBio, China). Dyes were stored in the dark and utilized after aliquoting. Dilute to the appropriate working concentration according to the instructions before use.

### 4.13 Confocal Microscopy

MARC‐145 cells were washed with phosphate‐buffered saline (PBS) (P1020, Beijing Solarbio Science & Technology Co., Ltd, China) following PRRSV infection and transfection treatment. An appropriate amount of dye was added for incubation, and after thorough washing, the cells were observed using a confocal microscope (Carl Zeiss LSM 880 Confocal Microscope).

### 4.14 Co‐Immunoprecipitation (Co‐IP)

HEK‐293T cells were seeded in a 10 cm culture dish and incubated overnight. The corresponding plasmids were transfected using TransIntro PL Transfection Reagent (FT301, TransGen Biotech, China). After 24 h, the cells were collected and lysed in Western and IP cell lysis buffer (P0013, Beyotime Biotech Inc., China). The lysate was centrifuged at 12 000 × g for 10 min, and the supernatant was incubated at room temperature for 1 h with anti‐HA, anti‐Flag, or anti‐c‐Myc magnetic beads (HY‐K0201, HY‐K0207, HY‐K0206, MedChem Express, USA). The precipitated complexes were collected using a magnetic stand, washed three times with lysis buffer, and eluted with loading buffer for immunoblot analysis.

### 4.15 UV–Vis Absorption Spectroscopy Analysis

Cells were collected, and the absorbance values of the samples at various wavelengths were measured using a NanoDrop spectrophotometer (ThermoFisher Scientific).

### 4.16 Aconitase (ACO) Activity Assay

Following cell collection, the Aconitase (ACO) Activity Assay Kit (BC4485, Beijing Solarbio Science & Technology Co., Ltd, China) was employed to extract mitochondrial ACO, cytoplasmic ACO, and total ACO from the cell samples. Absorbance was measured at 240 nm using a microplate reader, and the activities of mitochondrial ACO, cytoplasmic ACO, and total ACO in different samples were calculated based on protein concentration.

### 4.17 Cell Viability

Cell viability was evaluated using the Cell Counting Kit (CCK‐8) (6073212, Dakewe Biotech Co., Ltd, China). Cells were seeded in a 96‐well culture plate and cultured overnight. Following treatment with varying concentrations of the test substance for 48 h, 10 µL of CCK‐8 solution was added to each well for an additional 2 h of incubation. Absorbance was measured at 450 nm using a microplate reader.

### 4.18 L‐Carnitine Content Assay

Post‐treatment, the Human L‐Carnitine ELISA Kit (ELK9821, ELK Biotechnology, China) was utilized to measure absorbance at 450 nm using a microplate reader, and a standard curve was generated using Origin software to determine the concentration of L‐Carnitine in different samples.

### 4.19 LiP‐SMap

Cells were resuspended in PBS and homogenized to achieve complete lysis. The cell lysate containing approximately 1 mg of total protein was incubated with L‐Carnitine (20 µm) or DMSO at 25°C for 10 min. Following the addition of proteinase K, incubation continued for an additional 10 min, followed by heating at 98°C for 3 min. Sodium deoxycholate was added to a final concentration of 5%, and the mixture was heated at 98°C for 3 min to terminate the enzymatic reaction. The mixture was incubated at 37°C with 5 mm Tris (2‐carboxyethyl) phosphine hydrochloride for 40 min, followed by dark incubation at 25°C with 20 mm iodoacetamide for 30 min for alkylation. Trypsin digestion was performed at 37°C for 16 h. Formic acid was added to adjust the pH to below 2. The mixture was desalted using MonoSpin C18 (GL Sciences, Tokyo, Japan). The eluent was dried in a rotary evaporator and dissolved in 0.1% formic acid for mass spectrometry analysis. Differentially expressed proteins were analyzed applying a threshold of log2 fold change (log2FC) ≥ 1.5 and a *p*‐value < 0.05 to compare the L‐Carnitine treatment and control groups.

### 4.20 Molecular Docking

The protein 3D structure file was obtained from the Protein Data Bank (https://www.rcsb.org/), while the molecular structure of L‐Carnitine was sourced from PubChem. Molecular docking was conducted using AutoDock Vina 1.2.0, with visualization performed using software such as Discovery Studio Client and Pymol.

### 4.21 Cellular Thermal Shift Assay (CETSA)

Cell lysates from MARC‐145 cells were divided into two equal portions and treated with L‐Carnitine (20 µm) or DMSO for 1 h. The mixtures were subjected to heating at various temperatures (45°C, 50°C, 55°C, 60°C, 65°C, and 70°C) for 3 min. Following three freeze‐thaw cycles, samples were centrifuged at 12 000 rpm for 15 min at 4°C, and the supernatant was analyzed via Immunoblotting.

### 4.22 Dual Luciferase Reporter Assay

After co‐transfecting the reporter plasmid (containing a 1000 bp NCOA4 promoter fragment upstream of the ATG start codon) and reference plasmid for 24 h, cells were infected with PRRSV. The activities of firefly luciferase and Renilla luciferase were measured using the Dual‐Luciferase Reporter Assay System kit (E1910, Promega, United States of America). The relative fluorescence values for each sample were subsequently calculated.

### 4.23 Statistical Analysis

All data presented in this study were obtained from at least three biological replicates and are expressed as the mean ± standard deviation (SD). Statistical analyses were performed using two‐tailed Student's *t*‐tests, one‐way analysis of variance (ANOVA), or two‐way ANOVA. A *p*‐value of less than 0.05 (^*^) was considered statistically significant, while a *p*‐value of less than 0.01 (^**^) indicated a highly significant difference. The authors confirm that no artificial intelligence or AI‐assisted technologies were used in the design, analysis, or writing of this manuscript.

## Author Contributions

K.G., G.Z. and X.Z. designed the study and supervised the project. K.G., C.Y., Z.M., Y.W. and X.Z. performed the experiments and analyzed the data. K.G. and C.Y. wrote the original draft of the manuscript. C.H. participated in the writing and revision of the manuscript. K.G., C.Y., X.Z., and G.Z. reviewed and edited the manuscript. All authors have read and approved the final version of the manuscript.

## Funding

This study was supported by the National Natural Science Foundation of China (32302844, 32172699), the China Postdoctoral Science Foundation (2024M750114), and the National Key Research and Development Program of China (2023YFF1000901).

## Conflicts of Interest

All authors declare no conflicts of interest.

## Supporting information




**Supporting File 1**: advs75721‐sup‐0001‐SuppMat.docx.

**Supporting File 2**: advs75721‐sup‐0002‐FigureS9.docx.

**Supporting File 3**: advs75721‐sup‐0003‐TableS1‐S3.zip.

## Data Availability

The data that supports the findings of this study are available in the supplementary material of this article.

## References

[1] A. Allouch , A. David , S. M. Amie , et al., “p21‐mediated RNR2 Repression Restricts HIV‐1 Replication in Macrophages by Inhibiting dNTP Biosynthesis Pathway,” Proceedings of the National Academy of Sciences 110 (2013): E3997–4006. 10.1073/pnas.1306719110 PMC3801060 24082141

[2] N. Maio , B. A. P. Lafont , D. Sil , et al., “Fe‐S Cofactors in the SARS‐CoV‐2 RNA‐dependent RNA Polymerase Are Potential Antiviral Targets,” Science 373 (2021): 236–241. 34083449 10.1126/science.abi5224 PMC8892629

[3] Q. Ren , X. Xu , Z. Dong , et al., “Iron Deficiency Impairs Dendritic Cell Development and Function, Compromising Host Anti‐Infection Capacity,” Advanced Science 12 (2025): 2408348. 40305750 10.1002/advs.202408348 PMC12120711

[4] B. J. Leyshon , P. Ji , M. P. Caputo , S. M. Matt , and R. W. Johnson , “Dietary Iron Deficiency Impaired Peripheral Immunity but Did Not Alter Brain Microglia in PRRSV‐Infected Neonatal Piglets,” Frontiers in Immunology 9 (2018): 3150. 30778359 10.3389/fimmu.2018.03150 PMC6369153

[5] A. Ouyang , T. Chen , Y. Feng , et al., “The Hemagglutinin of Influenza A Virus Induces Ferroptosis to Facilitate Viral Replication,” Advanced Science 11 (2024): 2404365. 39159143 10.1002/advs.202404365 PMC11497066

[6] H. Drakesmith and A. Prentice , “Viral Infection and Iron Metabolism,” Nature Reviews Microbiology 6 (2008): 541–552. 18552864 10.1038/nrmicro1930

[7] L. Tong , J. Wang , Y. Ma , et al., “Viruses Hijack FPN1 to Disrupt Iron Withholding and Suppress Host Defense,” Nature Communications 16 (2025): 5912. 10.1038/s41467-025-60031-w PMC12216596 40595467

[8] A. Harrer , E. G. Meyron‐Holtz , and A. Meinhardt , “The Role of Iron in Normal and Impaired Testicular Function,” Andrology (2025): 1–15. 40464377 10.1111/andr.70068 PMC13665618

[9] X. Jin , C. Jiang , Z. Zou , et al., “Ferritinophagy in the Etiopathogenic Mechanism of Related Diseases,” The Journal of Nutritional Biochemistry 117 (2023): 109339. 37061010 10.1016/j.jnutbio.2023.109339

[10] W. X. Xu , X. Wen , Y. T. Fu , J. Yang , H. Cui , and R. F. Fan , “Key Role of Heavy Metals Toxicity,” Archives of Toxicology 99 (2025): 1257–1270. 39928088 10.1007/s00204-025-03963-y

[11] Y. Qu , W. Wang , M. Z. X. Xiao , Y. Zheng , and Q. Liang , “The Interplay between Lipid Droplets and Virus Infection,” Journal of Medical Virology 95 (2023): 28967. 10.1002/jmv.28967 37496184

[12] E. A. Monson , K. M. Crosse , M. Duan , et al., “Intracellular Lipid Droplet Accumulation Occurs Early Following Viral Infection and Is Required for an Efficient Interferon Response,” Nature Communications 12 (2021): 4303. 10.1038/s41467-021-24632-5 PMC8280141 34262037

[13] Z. Yuan , K. Cai , J. Li , et al., “ATG14 targets Lipid Droplets and Acts as an Autophagic Receptor for syntaxin18‐regulated Lipid Droplet Turnover,” Nature Communications 15 (2024): 631. 10.1038/s41467-024-44978-w PMC10799895 38245527

[14] C. Hilton , R. Sabaratnam , H. Drakesmith , and F. Karpe , “n Intertwined Relationship,” International Journal of Obesity 47 (2023): 554–563. 37029208 10.1038/s41366-023-01299-0 PMC10299911

[15] Z. Sun , Z. Ma , W. Cao , et al., “Calcium‐mediated Mitochondrial Fission and Mitophagy Drive Glycolysis to Facilitate Arterivirus Proliferation,” PLOS Pathogens 21 (2025): 1012872. 10.1371/journal.ppat.1012872 PMC11761150 39804926

[16] L. Wang , S. Xiao , J. Gao , et al., “Inhibition of Replication of Porcine Reproductive and respiratory Syndrome Virus by Hemin Is Highly Dependent on Heme Oxygenase‐1, but Independent of Iron in MARC‐145 Cells,” Antiviral Research 105 (2014): 39–46. 24583029 10.1016/j.antiviral.2014.02.010

[17] J. Li , Y. Zhou , W. Zhao , et al., “Porcine Reproductive and respiratory Syndrome Virus Degrades DDX10 via SQSTM1/p62‐dependent Selective Autophagy to Antagonize Its Antiviral Activity,” Autophagy 19 (2023): 2257–2274. 36779599 10.1080/15548627.2023.2179844 PMC10351467

[18] T. Tong , S. Deng , X. Zhang , L. Fang , J. Liang , and S. Xiao , “Inhibitory Effect and Mechanism of Gelatin Stabilized Ferrous Sulfide Nanoparticles on Porcine Reproductive and respiratory Syndrome Virus,” Journal of Nanobiotechnology 20 (2022): 70. 35123507 10.1186/s12951-022-01281-4 PMC8817501

[19] H. Zhang , F. Hu , O. Peng , et al., “Multi‐Omics Analysis by Machine Learning Identified Lysophosphatidic Acid as a Biomarker and Therapeutic Target for Porcine Reproductive and Respiratory Syndrome,” Advanced Science 11 (2024): 2402025. 38976572 10.1002/advs.202402025 PMC11425916

[20] B. Srour , S. Gervason , M. H. Hoock , et al., “Iron Insertion at the Assembly Site of the ISCU Scaffold Protein Is a Conserved Process Initiating Fe–S Cluster Biosynthesis,” Journal of the American Chemical Society 144 (2022): 17496–17515. 36121382 10.1021/jacs.2c06338 PMC10163866

[21] A. Villalta , B. Srour , A. Lartigue , et al., “Evidence for [2Fe‐2S]^2+^ and Linear [3Fe‐4S]^1+^ Clusters in a Unique Family of Glycine/Cysteine‐Rich Fe‐S Proteins from Megavirinae Giant Viruses,” Journal of the American Chemical Society 145 (2023): 2733–2738. 36705935 10.1021/jacs.2c10484

[22] K. Gari , A. M. León Ortiz , V. Borel , H. Flynn , J. M. Skehel , and S. J. Boulton , “MMS19 links Cytoplasmic Iron‐sulfur Cluster Assembly to DNA Metabolism,” Science 337 (2012): 243–245. 22678361 10.1126/science.1219664

[23] B. Uner , A. D. Ergin , I. A. Ansari , M. S. Macit‐Celebi , S. A. Ansari , and H. M. A. Kahtani , “Assessing the in Vitro and in Vivo Performance of L‐Carnitine‐Loaded Nanoparticles in Combating Obesity,” Molecules 28 (2023): 7115. 37894594 10.3390/molecules28207115 PMC10609287

[24] K. Smolková and K. Gotvaldová , “n Emerging Perspective,” International Journal of Biological Sciences 21 (2025): 1863–1873. 40083687 10.7150/ijbs.105361 PMC11900811

[25] P.‐P. Liu , J. Liu , W.‐Q. Jiang , et al., “Elimination of Chronic Lymphocytic Leukemia Cells in Stromal Microenvironment by Targeting CPT with an Antiangina Drug Perhexiline,” Oncogene 35 (2016): 5663–5673. 27065330 10.1038/onc.2016.103 PMC5064824

[26] R. Kang , G. Ji , X. Yang , et al., “Investigation on Host Susceptibility of Tibetan Pig to Infection of Porcine Reproductive and respiratory Syndrome Virus through Viral Challenge Study,” Veterinary Microbiology 183 (2016): 62–68. 26790936 10.1016/j.vetmic.2015.11.035

[27] W. Liang , Z. Li , P. Wang , et al., “Differences of Immune Responses Between Tongcheng (Chinese Local Breed) and Large White Pigs after Artificial Infection with Highly Pathogenic Porcine Reproductive and Respiratory Syndrome Virus,” Virus Research 215 (2016): 84–93. 26878768 10.1016/j.virusres.2016.02.004

[28] P. Zhou , S. Zhai , X. Zhou , et al., “Molecular Characterization of Transcriptome‐wide Interactions Between Highly Pathogenic Porcine Reproductive and respiratory Syndrome Virus and Porcine Alveolar Macrophages in Vivo,” International Journal of Biological Sciences 7 (2011): 947–959. 21850204 10.7150/ijbs.7.947 PMC3157269

[29] Q. Wu , Y. Han , X. Wu , et al., “Integrated Time‐series Transcriptomic and Metabolomic Analyses Reveal Different Inflammatory and Adaptive Immune Responses Contributing to Host Resistance to PRRSV,” Frontiers in Immunology 13 (2022): 960709. 36341362 10.3389/fimmu.2022.960709 PMC9631489

[30] J. He , Z. Li , P. Xia , et al., “Ferroptosis and Ferritinophagy in Diabetes Complications,” Molecular Metabolism 60 (2022): 101470. 35304332 10.1016/j.molmet.2022.101470 PMC8980341

[31] Y. Sun , Q. Bao , B. Xuan , et al., “Human Cytomegalovirus Protein pUL38 Prevents Premature Cell Death by Binding to Ubiquitin‐Specific Protease 24 and Regulating Iron Metabolism,” Journal of Virology 92 (2018): 13. 10.1128/JVI.00191-18 PMC6002719 29695420

[32] K. Ohta , N. Saka , and M. Nishio , “Human Parainfluenza Virus Type 2 V Protein Modulates Iron Homeostasis,” Journal of Virology 95 (2021): 6. 10.1128/JVI.01861-20 PMC8094939 33408172

[33] O. Stehling , A. A. Vashisht , J. Mascarenhas , et al., “MMS19 assembles Iron‐sulfur Proteins Required for DNA Metabolism and Genomic Integrity,” Science 337 (2012): 195–199. 22678362 10.1126/science.1219723 PMC3420340

[34] N. Santana‐Codina , M. Q. del Rey , K. S. Kapner , et al., “NCOA4‐Mediated Ferritinophagy Is a Pancreatic Cancer Dependency via Maintenance of Iron Bioavailability for Iron–Sulfur Cluster Proteins,” Cancer Discovery 12 (2022): 2180–2197. 35771492 10.1158/2159-8290.CD-22-0043 PMC9437572

[35] S. W. Alvarez , V. O. Sviderskiy , E. M. Terzi , et al., “NFS1 undergoes Positive Selection in Lung Tumours and Protects Cells from Ferroptosis,” Nature 551 (2017): 639–643. 29168506 10.1038/nature24637 PMC5808442

[36] M. S. Petronek , D. R. Spitz , and B. G. Allen , “Iron‐Sulfur Cluster Biogenesis as a Critical Target in Cancer,” Antioxidants 10 (2021): 1458. 34573089 10.3390/antiox10091458 PMC8465902

[37] R. Lill and S. A. Freibert , “Mechanisms of Mitochondrial Iron‐Sulfur Protein Biogenesis,” Annual Review of Biochemistry 89 (2020): 471–499. 10.1146/annurev-biochem-013118-111540 31935115

[38] A. Pandey , J. Pain , A. K. Ghosh , A. Dancis , and D. Pain , “Fe‐S Cluster Biogenesis in Isolated Mammalian Mitochondria,” Journal of Biological Chemistry 290 (2015): 640–657. 25398879 10.1074/jbc.M114.610402 PMC4281764

[39] Y. Chen , G. H. Cai , B. Xia , et al., “Mitochondrial Aconitase Controls Adipogenesis through Mediation of Cellular ATP Production,” The FASEB Journal 34 (2020): 6688–6702. 32212192 10.1096/fj.201903224RR

[40] M. Martin‐Perez , U. Urdiroz‐Urricelqui , C. Bigas , and S. A. Benitah , “The Role of Lipids in Cancer Progression and Metastasis,” Cell Metabolism 34 (2022): 1675–1699. 36261043 10.1016/j.cmet.2022.09.023

[41] D. R. Crooks , N. Maio , A. N. Lane , et al., “Acute Loss of Iron–sulfur Clusters Results in Metabolic Reprogramming and Generation of Lipid Droplets in Mammalian Cells,” Journal of Biological Chemistry 293 (2018): 8297–8311. 29523684 10.1074/jbc.RA118.001885 PMC5971457

[42] C. Yuan , K. Guan , and G. Zhang , “STEAP3 Inhibits Porcine Reproductive and Respiratory Syndrome Virus Replication by Regulating Fatty Acid and Lipid Droplet Synthesis,” Vet Sci 12 (2025): 147. 40005907 10.3390/vetsci12020147 PMC11861627

[43] C. Shao , Z. Yu , T. Luo , et al., “Chitosan‐Coated Selenium Nanoparticles Attenuate PRRSV Replication and ROS/JNK‐Mediated Apoptosis in Vitro,” International Journal of Nanomedicine 17 (2022): 3043–3054. 35832119 10.2147/IJN.S370585 PMC9273186

[44] C. Zhou , G. Bao , and Y. Chen , “TRIM46 accelerates H1N1 Influenza Virus‐induced Ferroptosis and Inflammatory Response by Regulating SLC7A11 Ubiquitination,” Journal of Bioenergetics and Biomembranes 56 (2024): 631–643. 39531094 10.1007/s10863-024-10043-w

[45] J. D. Hulse , S. R. Ellis , and L. M. Henderson , “Carnitine Biosynthesis. Beta‐Hydroxylation of Trimethyllysine by an Alpha‐ketoglutarate‐dependent Mitochondrial Dioxygenase,” Journal of Biological Chemistry 253 (1978): 1654–1659. 627563

[46] F. M. Vaz and R. J. Wanders , “Carnitine Biosynthesis in Mammals,” Biochemical Journal 361 (2002): 417–429. 11802770 10.1042/0264-6021:3610417 PMC1222323

[47] S. Hu , X. Liang , Y. Qin , et al., “Alnustone Ameliorates Metabolic Dysfunction‐Associated Steatotic Liver Disease by Facilitating Mitochondrial Fatty Acid β‐Oxidation via Targeting Calmodulin,” Advanced Science 12 (2025): 11984. 10.1002/advs.202411984 PMC12376512 40470949

[48] M. U. Anwar , O. A. Sergeeva , L. Abrami , et al., “ER‐Golgi‐localized Proteins TMED2 and TMED10 Control the Formation of Plasma Membrane Lipid Nanodomains,” Developmental Cell 57 (2022): 2334–2346.e8. 36174556 10.1016/j.devcel.2022.09.004

[49] J. Yuan , T. Lv , J. Yang , et al., “HDLBP‐stabilized lncFAL Inhibits Ferroptosis Vulnerability by Diminishing Trim69‐dependent FSP1 Degradation in Hepatocellular Carcinoma,” Redox Biology 58 (2022): 102546. 36423520 10.1016/j.redox.2022.102546 PMC9692041

[50] R. Tan , X. Xu , W. Hong , and T. Wang , “Analysis of Biogenesis of Lipid Droplets by Examining Rab40c Associating with Lipid Droplets,” Methods in Molecular Biology 1270 (2015): 125–135. 25702114 10.1007/978-1-4939-2309-0_10

[51] Y. Miyanari , K. Atsuzawa , N. Usuda , et al., “The Lipid Droplet Is an Important Organelle for hepatitis C Virus Production,” Nature Cell Biology 9 (2007): 1089–1097. 17721513 10.1038/ncb1631

[52] M. M. Samsa , J. A. Mondotte , N. G. Iglesias , et al., “Dengue Virus Capsid Protein Usurps Lipid Droplets for Viral Particle Formation,” PLoS Pathogens 5 (2009): 1000632. 10.1371/journal.ppat.1000632 PMC2760139 19851456

[53] T. Yang , N. Liang , J. Zhang , et al., “OCTN_2_ Enhances PGC‐1α‐mediated Fatty Acid Oxidation and OXPHOS to Support Stemness in Hepatocellular Carcinoma,” Metabolism 147 (2023): 155628. 37315888 10.1016/j.metabol.2023.155628

[54] Z. Ma , L. Guo , R. Ji , et al., “Pseudorabies Virus Induces Ferroptosis by Disrupting Iron Homeostasis through Activation of TfR1 and Ferritinophagy,” Journal of Virology 99 (2025): 0097425. 10.1128/jvi.00974-25 PMC12456129 40891826

[55] W. Zhang , K. Chen , Y. Guo , Y. Chen , and X. Liu , “Involvement of PRRSV NSP_3_ and NSP_5_ in the Autophagy Process,” Virology Journal 16 (2019): 13. 30691473 10.1186/s12985-019-1116-x PMC6350329

[56] H. Dong , Q. Pei , J. Ren , et al., “Cholesterol‐dependent Nsp_5_‐endosomes co‐trafficking to Lysosomes Facilitates Porcine Reproductive and respiratory Syndrome Virus Replication by Activating Autophagy,” Veterinary Microbiology 305 (2025): 110507. 40215803 10.1016/j.vetmic.2025.110507

[57] M. Shariq , N. Quadir , A. Alam , et al., “The Exploitation of Host Autophagy and Ubiquitin Machinery by Mycobacterium Tuberculosis in Shaping Immune Responses and Host Defense during Infection,” Autophagy 19 (2023): 3–23. 35000542 10.1080/15548627.2021.2021495 PMC9809970

[58] D. L. Haakonsen and M. Rape , “Branching out: Improved Signaling by Heterotypic Ubiquitin Chains,” Trends in Cell Biology 29 (2019): 704–716. 31300189 10.1016/j.tcb.2019.06.003

