# Supplementary material for: Ferritinophagy Rewires Carnitine‐Dependent Lipid Metabolism to Inhibit PRRSV and IAV Replication
Source: Adv Sci (Weinh). 2026 May 20:e75721. Online ahead of print. doi: 10.1002/advs.75721 (PMC13335848; doi:10.1002/advs.75721)
Supplement: Supplementary file 1 — Supporting File 1: advs75721‐sup‐0001‐SuppMat.docx. [file ADVS-9999-e75721-s001.docx]

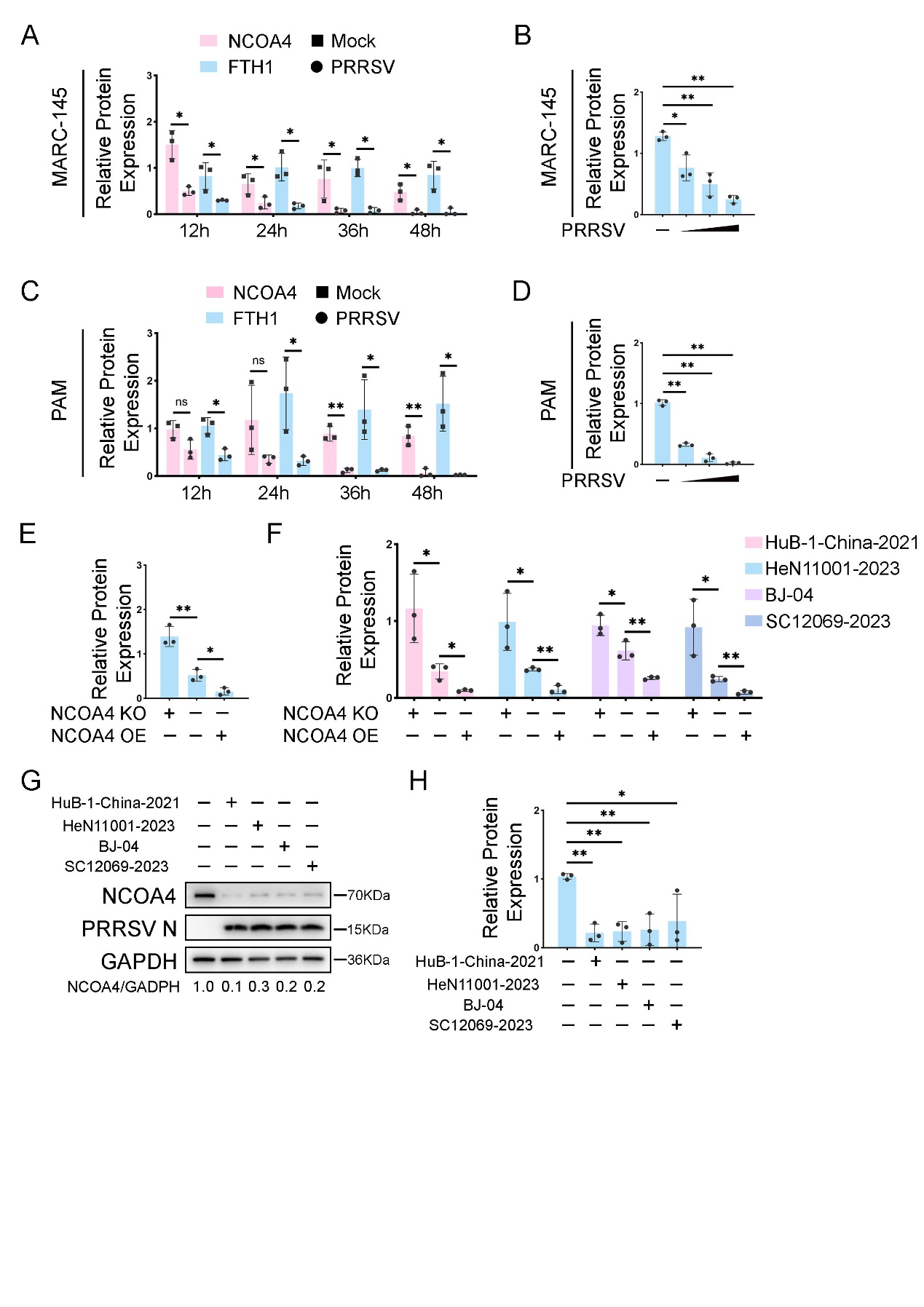


**Figure S1. PRRSV suppresses the expression of NCOA4.** (A-F) Quantification of relative protein expression from Figure 1A–D, F and H. ns indicates not significant, **P* < 0.05, ***P* < 0.01, Student's t-test, n = 3. (G) (H) MARC-145 cells were infected with HP-PRRSV (HuB-1-China-2021), NADC30-like (HeN11001-2023), and VR2332 (BJ-04, SC12069-2023). Cells were harvested 24 h post-infection. (G) Immunoblotting was performed to detect the expression of NCOA4 and PRRSV N protein. (Original blot images in Figure S9 s1a). (H) Quantification of relative protein expression. **P* < 0.05, ***P* < 0.01, Student's t-test, n = 3.


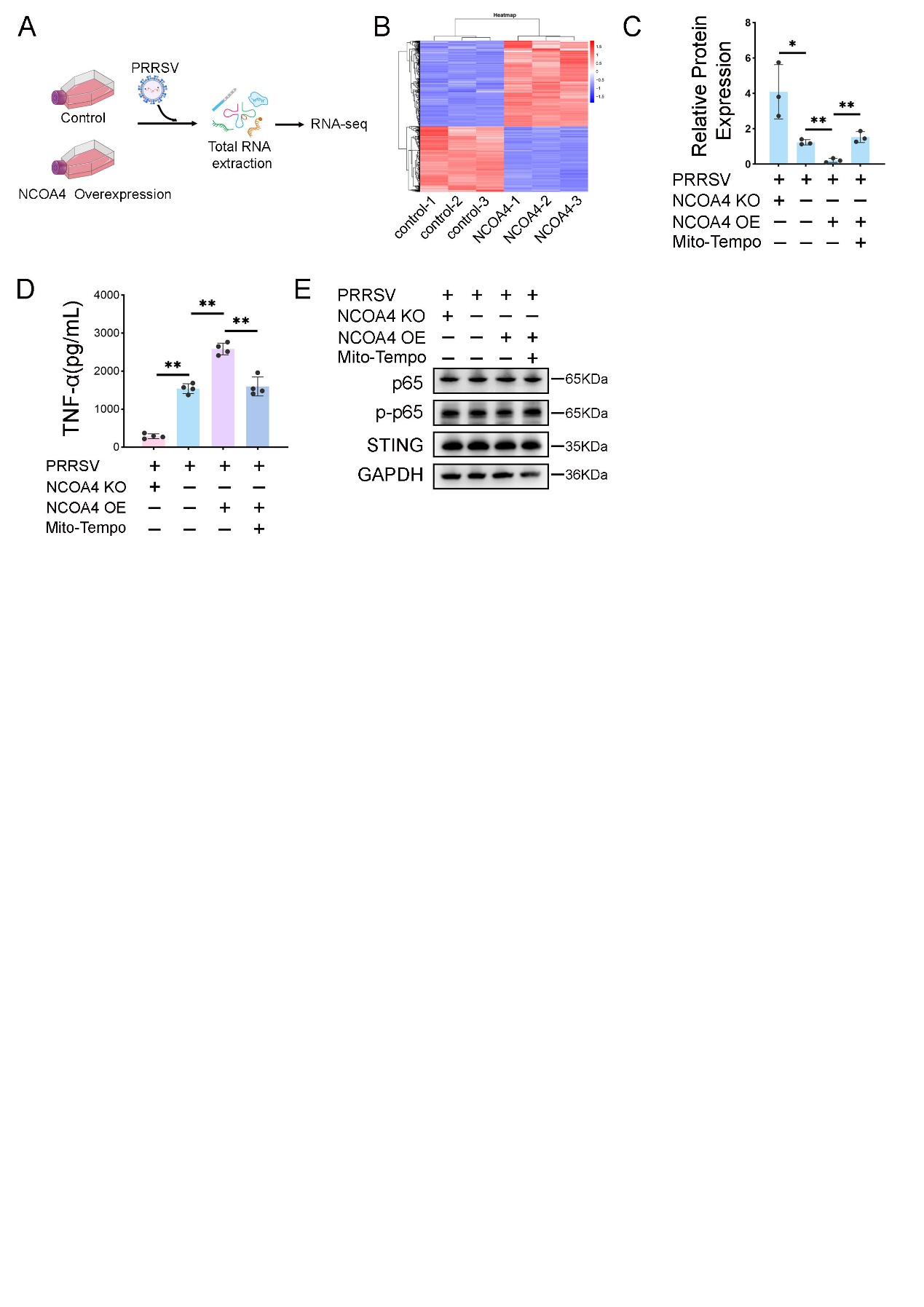


**Figure S2. NCOA4 inhibits PRRSV replication by remodeling mitochondria.** (A) Workflow for transcriptome sequencing. (B) Heatmap displaying differentially expressed genes. (C) Quantification of relative protein expression from Figure 2J. **P* < 0.05, ***P* < 0.01, Student's t-test, n = 3. (D) TNF-α levels were measure, with consistent cell treatments, reported as pg/mL. ***P* < 0.01, one-way ANOVA, n ≥ 3. (E) Immunoblotting analysis was also performed to evaluate the expression of p65, p-p65, and STING protein, with consistent cell treatments. (Original blot images in Figure S9 s2a).


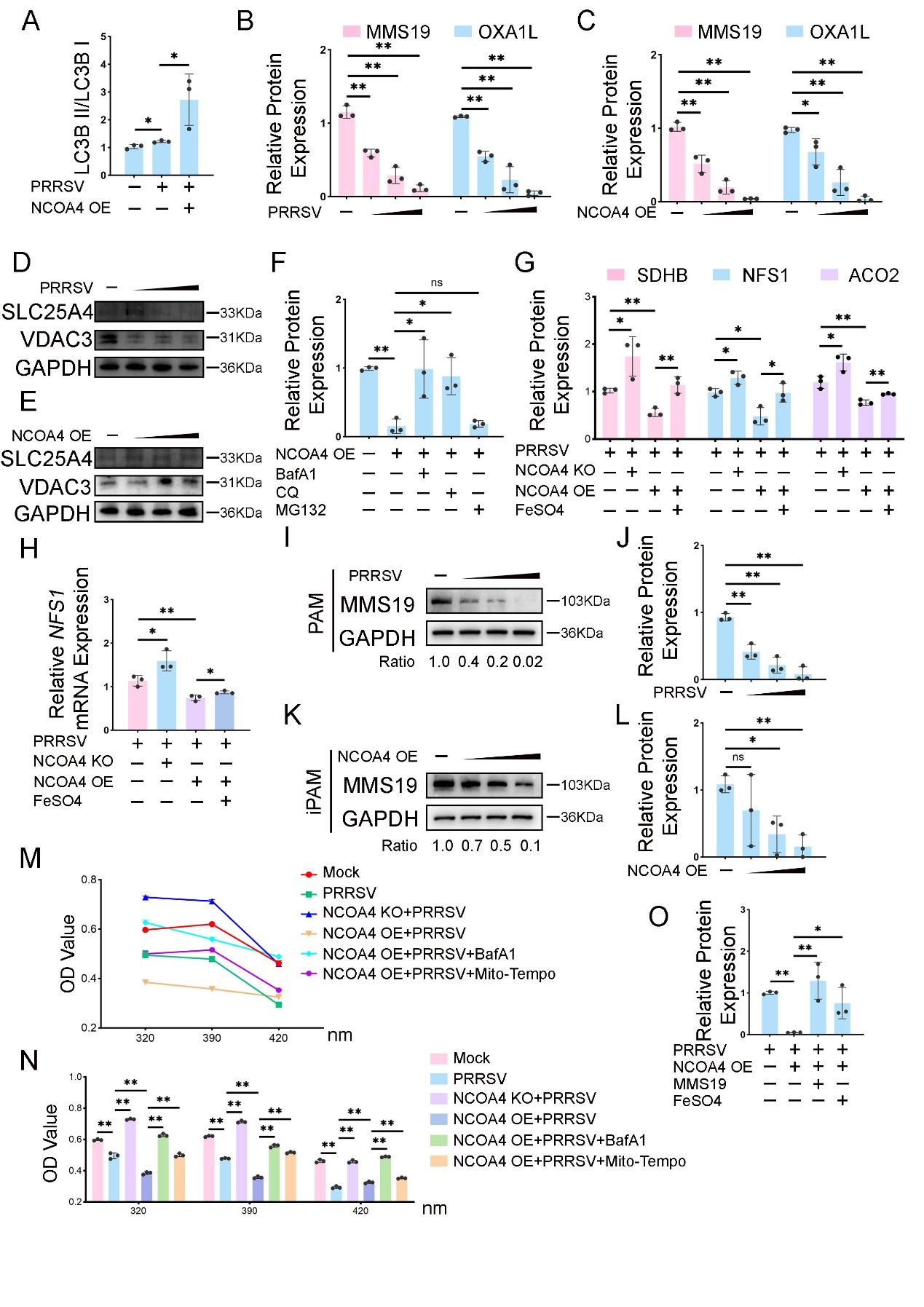


**Figure S3. NCOA4-mediated ferritinophagy inhibits the replication of PRRSV through the disruption of iron-sulfur proteins formation.** (A-C) (F-G) (O) Quantification of relative protein expression from the corresponding immunoblotting experiments shown in the related main Figure 3. **P* < 0.05, ***P* < 0.01, Student's t-test, n = 3. (D) (E) Immunoblotting analysis was also performed to evaluate the expression of SLC25A4 and VDAC3 protein. (Original blot images in Figure S9 s3a- s3b). (H) MARC-145 cells were transfected to overexpress NCOA4, and FeSO_4_ was added 12 h prior to sample collection. RT-qPCR was utilized to quantify *NFS1* mRNA levels, normalized to the reference gene GAPDH. **P* < 0.05, ***P* < 0.01, Student's t-test, n = 3. (I) (J) PAM cells were infected with PRRSV at various MOI, and cells were collected 24 h post-infection. (I) Immunoblotting analysis was conducted to evaluate the expression levels of MMS19 protein. (Original blot images in Figure S9 s3c). (J) Quantification of relative protein expression. ***P* < 0.01, Student's t-test, n = 3. (K) (L) A NCOA4 recombinant plasmid was transfected into iPAM cells in a concentration gradien. (K) Immunoblotting analysis to assess MMS19 protein levels. (Original blot images in Figure S9 s3d). (L) Quantification of relative protein expression. ns indicates not significant, **P* < 0.05, ***P* < 0.01, Student's t-test, n = 3. (M) MARC-145 cells with NCOA4 KO, NCOA4 OE, and WT were infected with PRRSV, and BafA1 and Mito-Tempo were added 12 h prior to sample collection to measure absorbance values at different wavelengths. (N) Statistical analysis of absorbance values at different wavelengths. ***P* < 0.01, Student's t-test, n = 3.


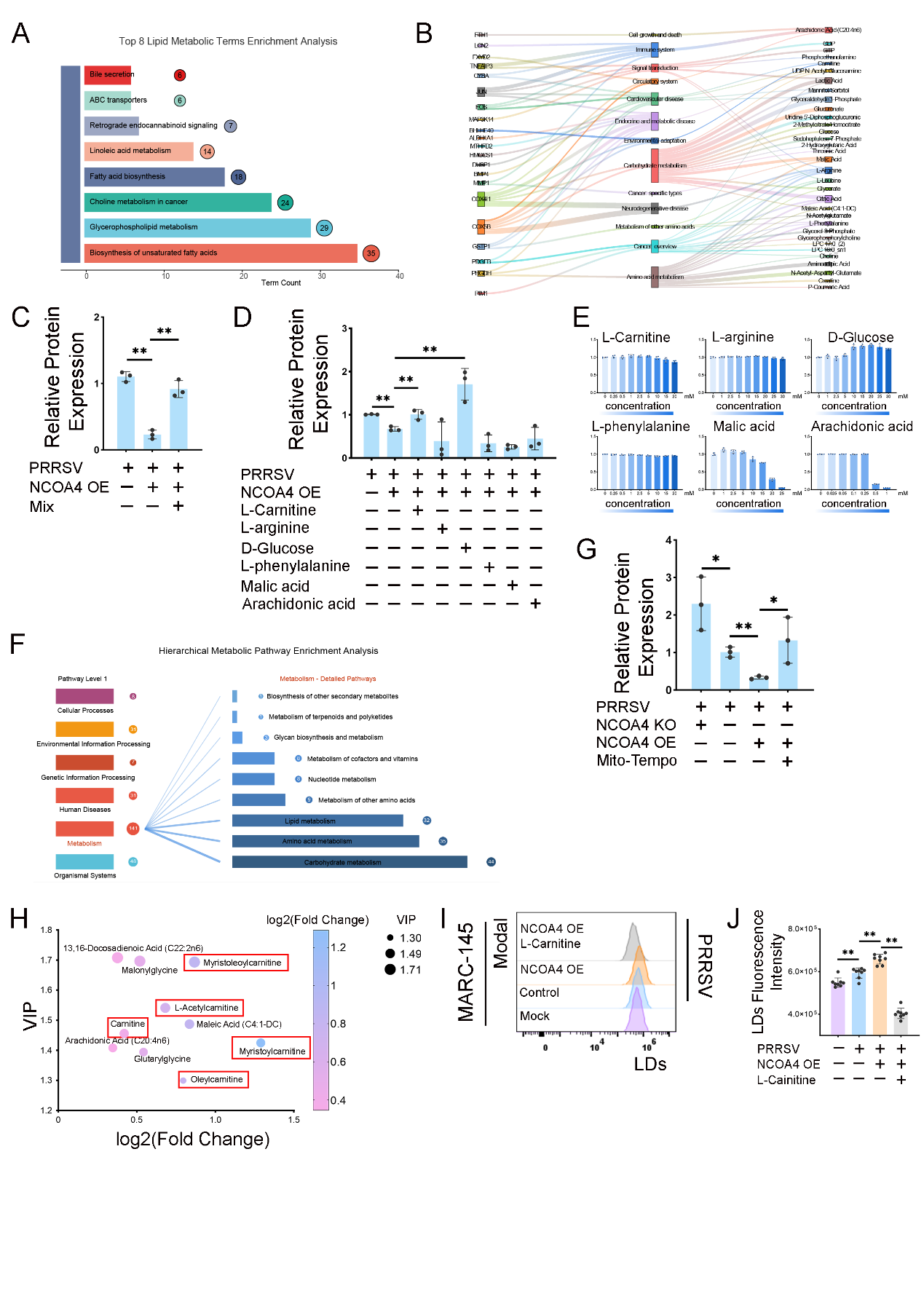


**Figure S4. NCOA4 facilitates the biogenesis of lipid droplets by reprogramming lipid metabolism.** (A) Lipid Metabolic Terms Enrichment Analysis. (B) Combined analysis of transcriptomic and metabolomic. (C) (D) (G) Quantification of relative protein expression from Figure 4C, D and J. ***P* < 0.01, Student's t-test, n = 3. (E) Addition of different concentrations of metabolite small molecules to MARC-145 cells, using CCK-8 for cytotoxicity detection. (F) Hierarchical Metabolic Pathway Enrichment Analysis. (H) Analysis of DEMs. (I) Flow cytometry analysis of LDs levels in MARC-145 cells, with quantitative measurement based on average fluorescence intensity (J) ***P* < 0.01, one-way ANOVA, n ≥ 3.


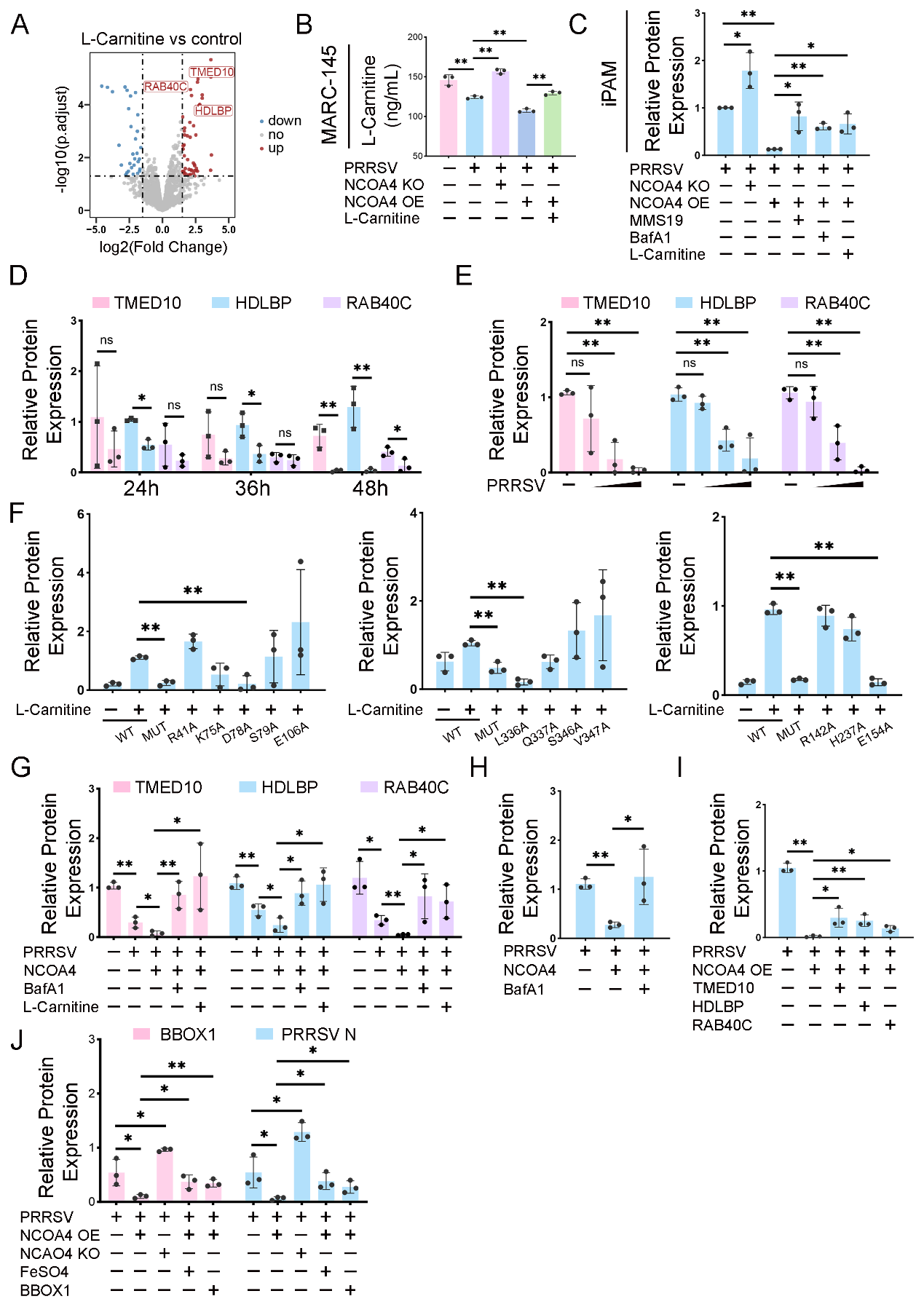


**Figure S5. Interaction mechanism between L-Carnitine and TMED10, HDLBP, or RAB40C.** (A) Volcano plot. (B) The intracellular L-Carnitine content in MARC-145 cells was detected using an L-Carnitine detection kit, ***P* < 0.01, one-way ANOVA, n=3. (C-J) Quantification of relative protein expression from Figure 5B, E, F and I-M. ns indicates not significant, **P* < 0.05, ***P* < 0.01, Student's t-test, n = 3.


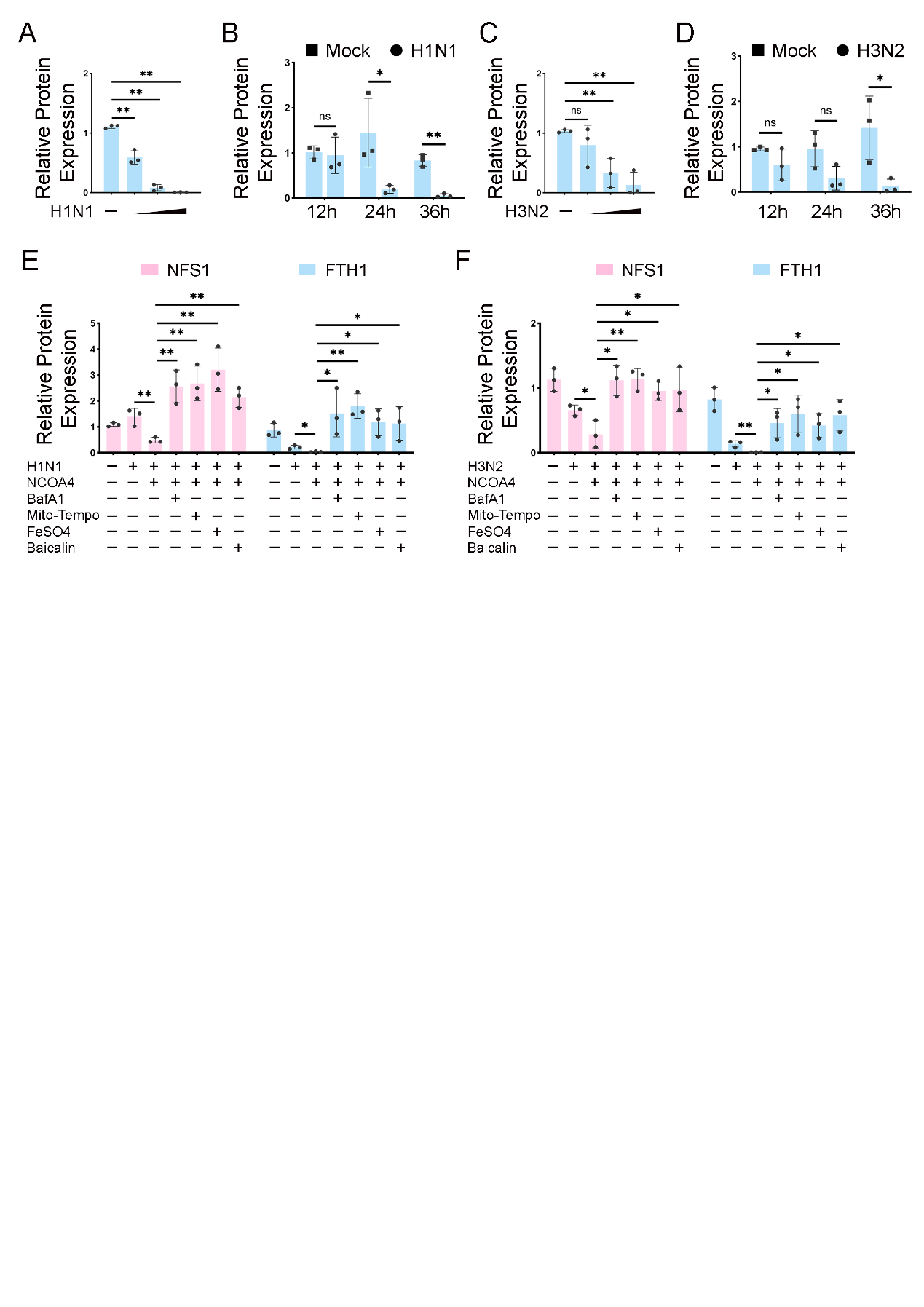


**Figure S6. Inhibitory effect of the NCOA4-mediated iron-lipid metabolism axis on H1N1 and H3N2 replication.** (A-F) Quantification of relative protein expression from Figure 6C-F, K and L. ns indicates not significant, **P* < 0.05, ***P* < 0.01, Student's t-test, n = 3.


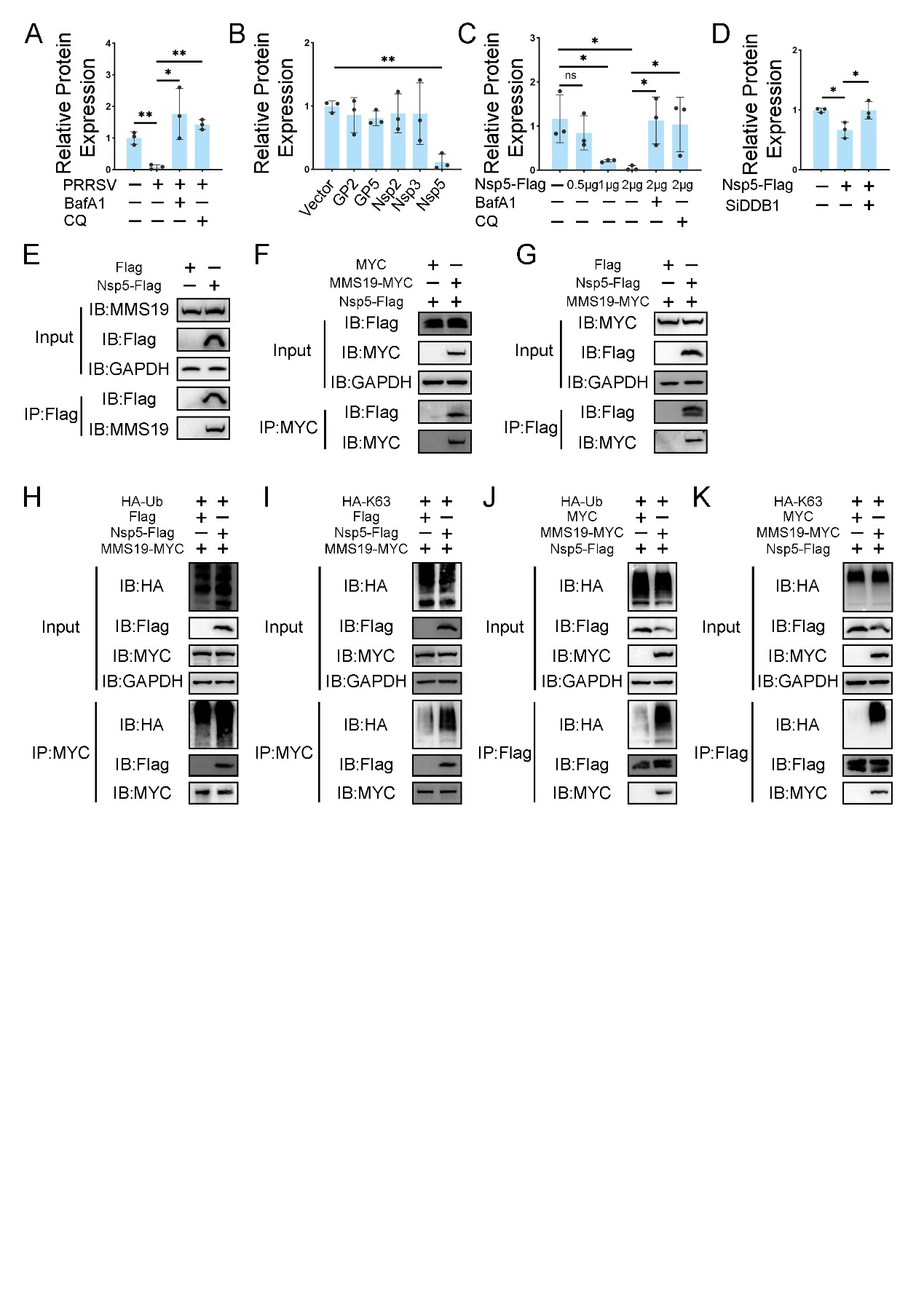


**Figure S7. Interaction mechanism between PRRSV Nsp5 and MMS19.** (A-D) Quantification of relative protein expression from Figure 7B-D and F. ns indicates not significant, **P* < 0.05, ***P* < 0.01, Student's t-test, n = 3. (E) Flag empty vector and Nsp5-Flag recombinant plasmids were transfected into HEK-293T cells, and cell lysates were collected 36 h post-transfection for immunoblotting analysis. (Original blot images in Figure S9 s7a). (F) (G) HEK-293T cells were transfected with MMS19-MYC and Nsp5-Flag or the corresponding empty vector for 36 h, followed by affinity separation using anti-MYC or anti-Flag beads, and co-immunoprecipitation analysis with specific antibodies. (Original blot images in Figure S9 s7b-s7c). (H-K) HEK-293T cells were co-transfected with Flag empty vector or Nsp5-Flag and MMS19-MYC along with HA-Ub or HA-K63, followed by affinity separation using anti-MYC or anti-Flag beads and co-immunoprecipitation analysis with specific antibodies. (Original blot images in Figure S9 s7d-s7g).


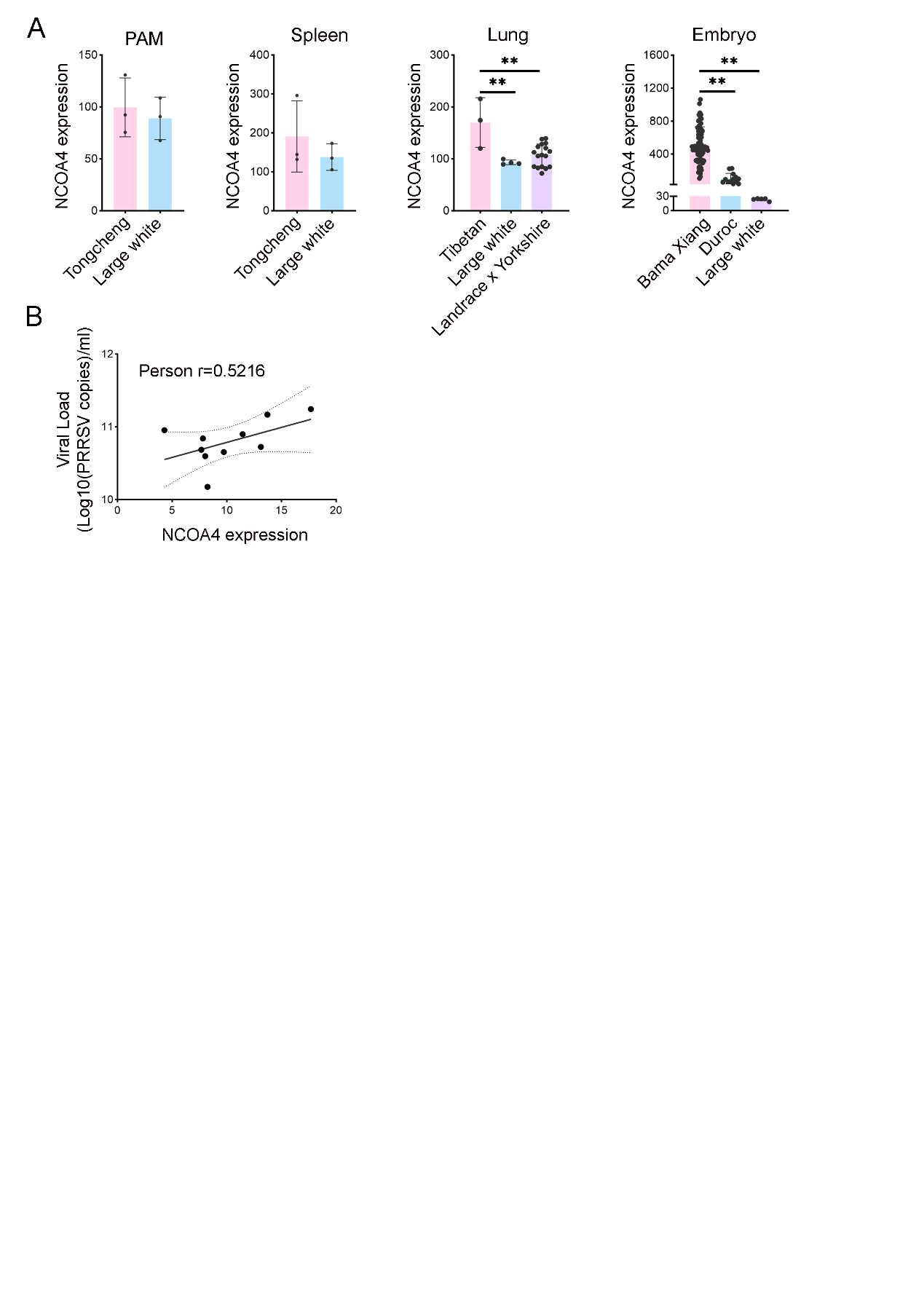


**Figure S8. The correlation between NCOA4 expression levels and the viral load of PRRSV *in vivo*.** (A) NCOA4 expression in the PAM, spleen, lung, and embryo of PRRSV-resistant and PRRSV-sensitive pig breeds. ***P* < 0.01, one-way ANOVA, n ≥ 3. (B) Correlation analysis of NCOA4 expression and viral load during PRRSV infection, n = 10.
