# Supplementary material for: Ferritinophagy Rewires Carnitine‐Dependent Lipid Metabolism to Inhibit PRRSV and IAV Replication
Source: Adv Sci (Weinh). 2026 May 20:e75721. Online ahead of print. doi: 10.1002/advs.75721 (PMC13335848; doi:10.1002/advs.75721)

**Figure S9. Original, unprocessed immunoblot images for all Western blot data presented in the main and supplementary figures.**

This file contains the complete, uncropped scans of all blots used in this study. Each image is labeled with the corresponding figure and panel identifier (e.g., 1a, 1b). Molecular weight markers (in kDa) are indicated for each blot. All images are provided without any adjustments to brightness or contrast, except for the application of uniform linear adjustments to the entire image for clarity of presentation.

**Related to Figures 1, 2, 3, 4, 5, 6, 7 and supplementary figures.**


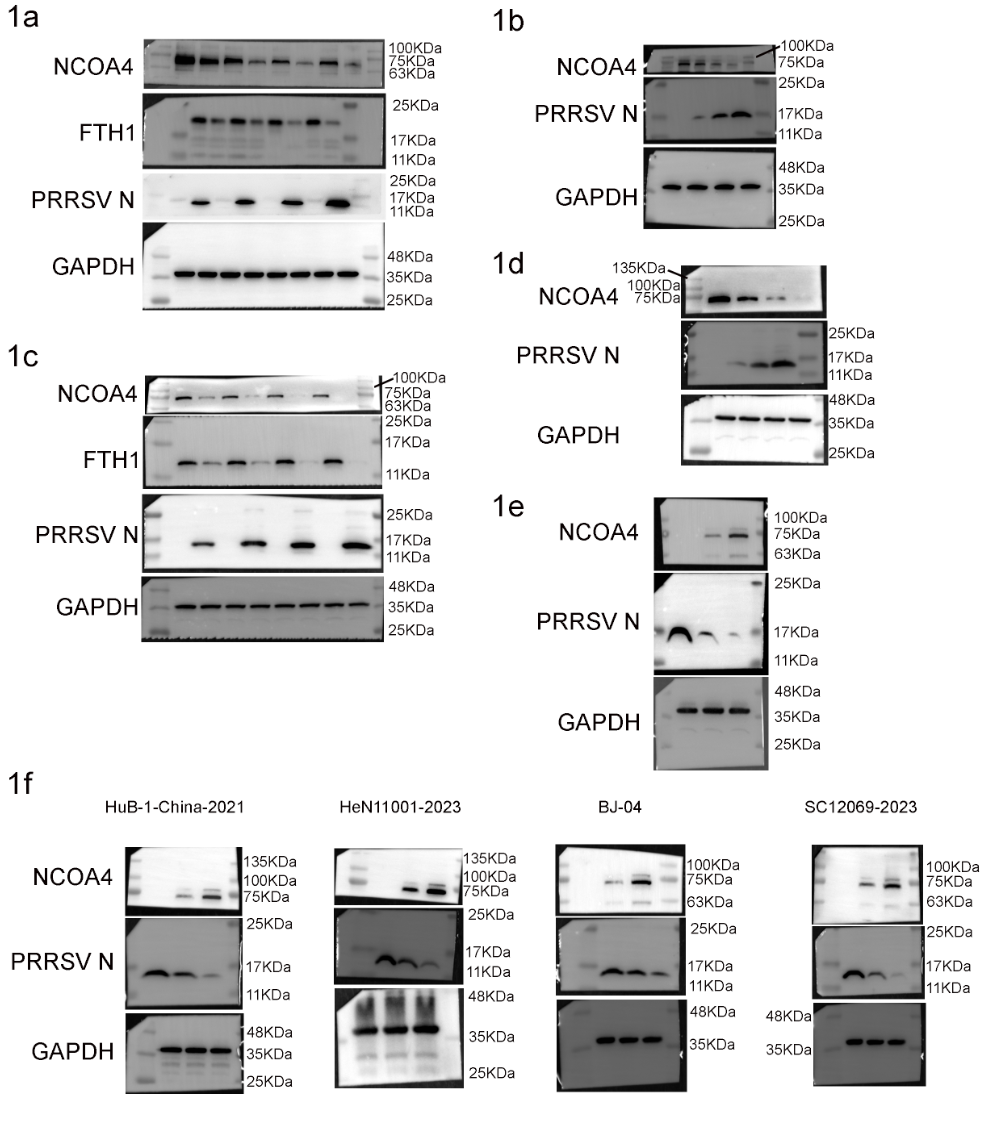


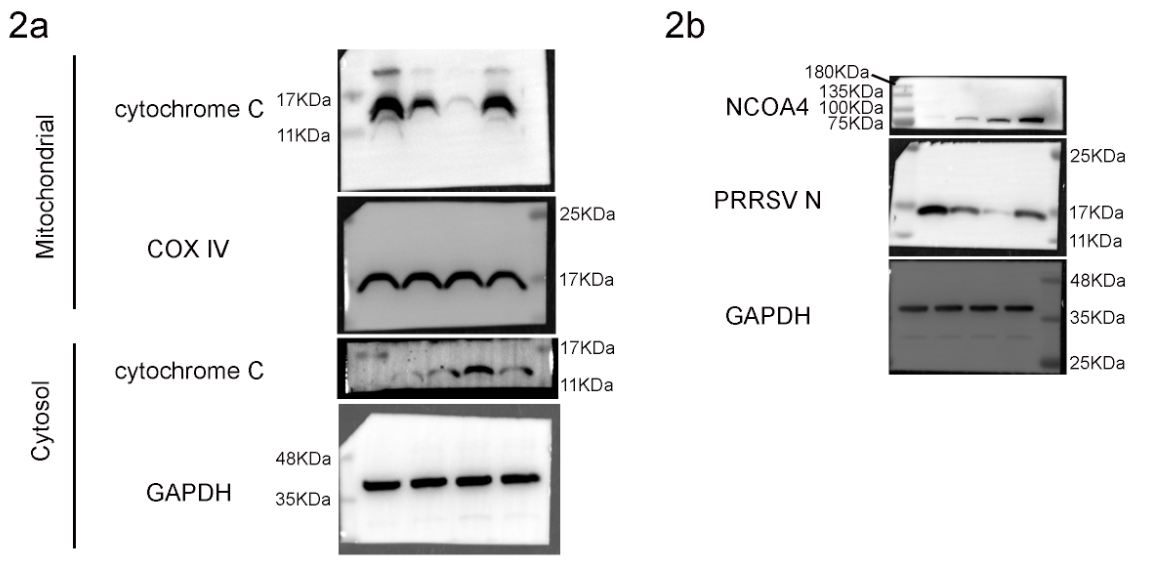


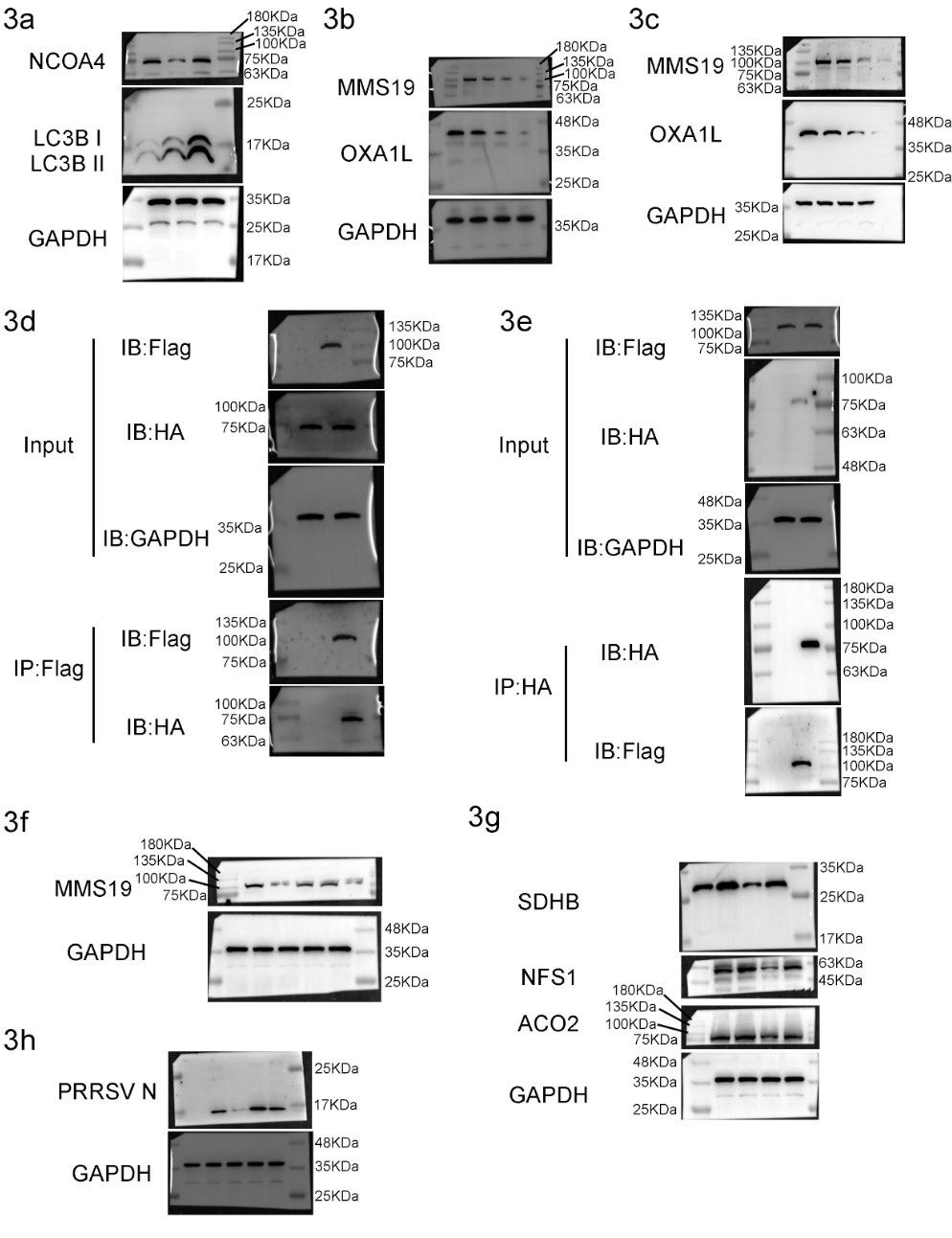


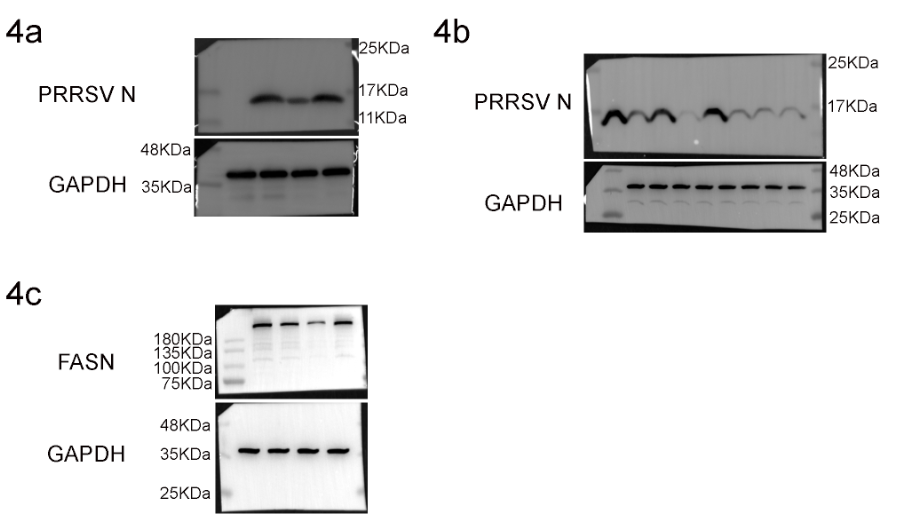


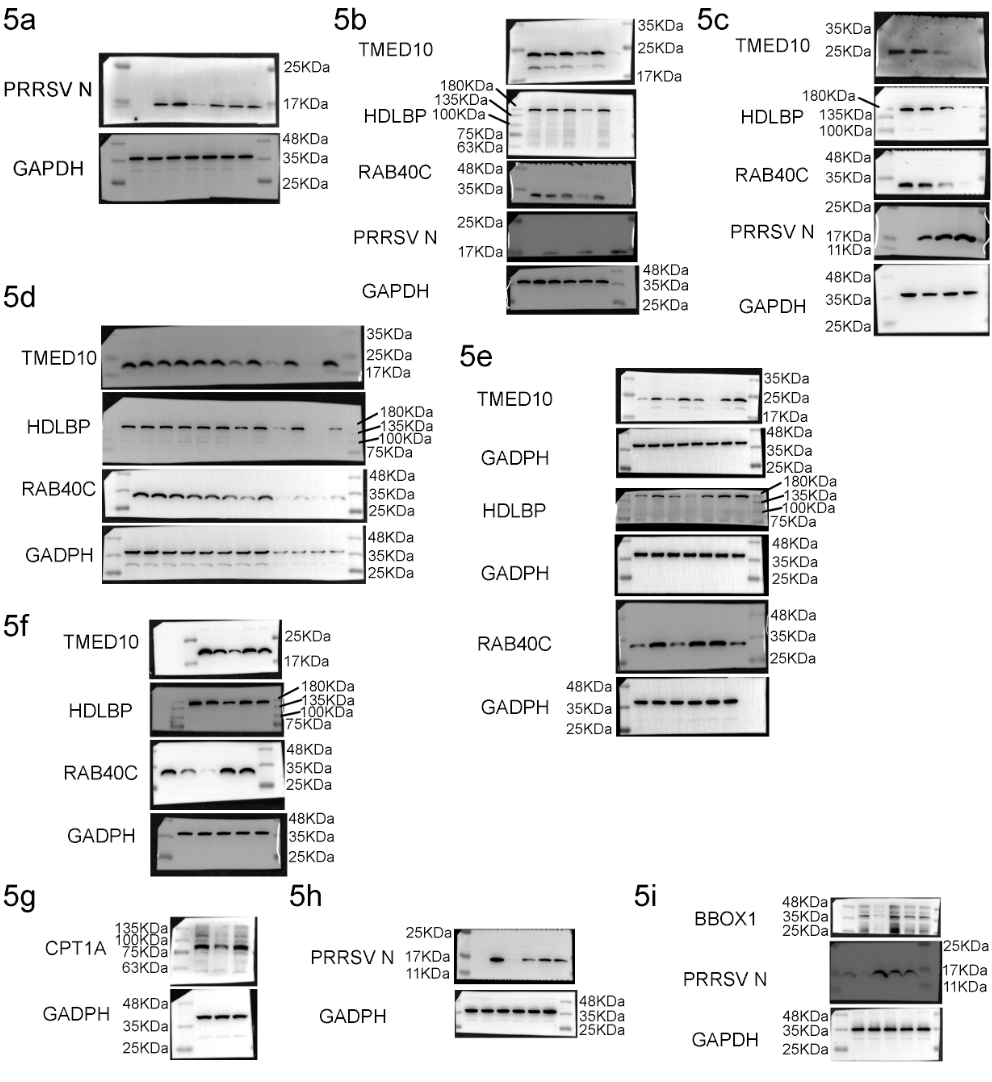


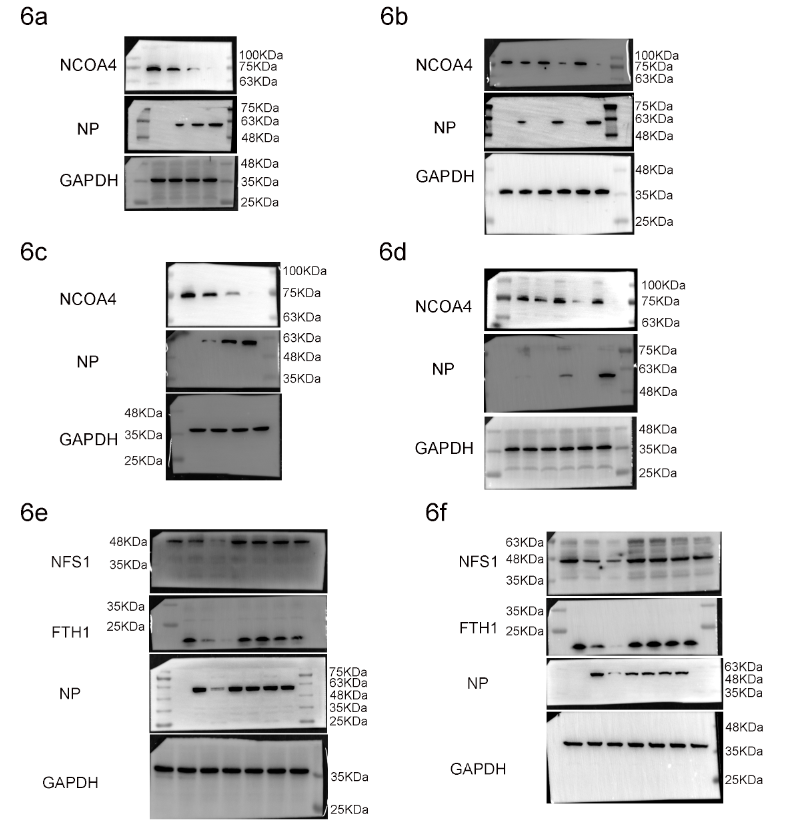


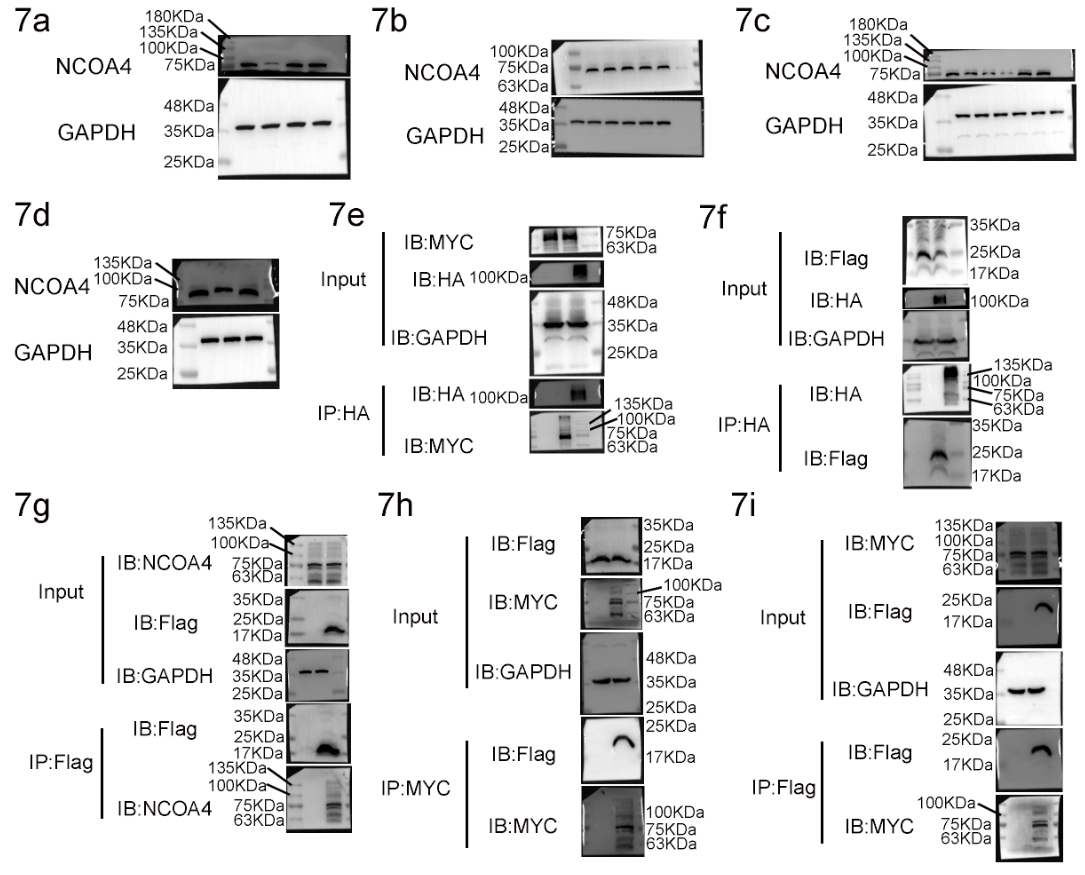


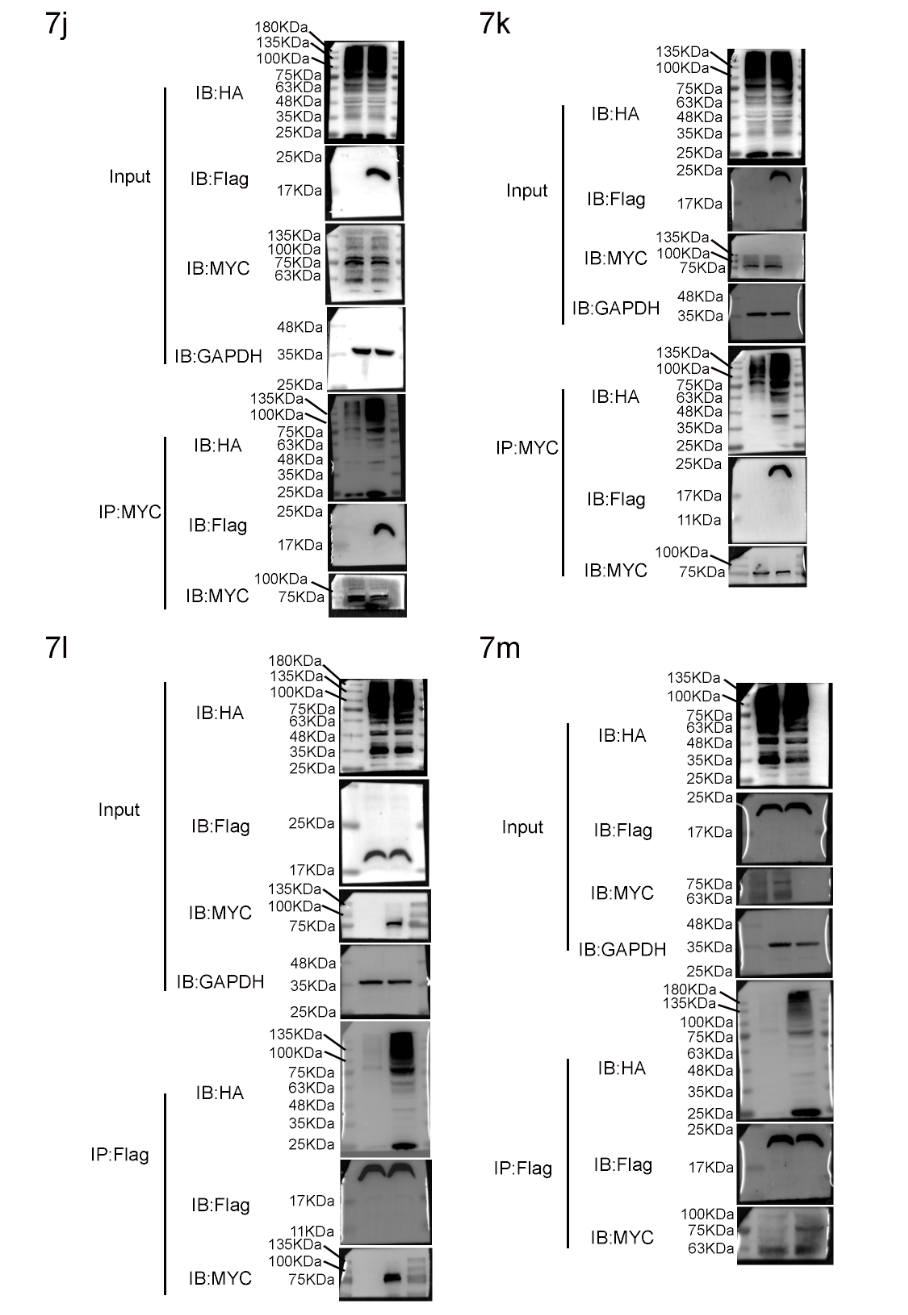


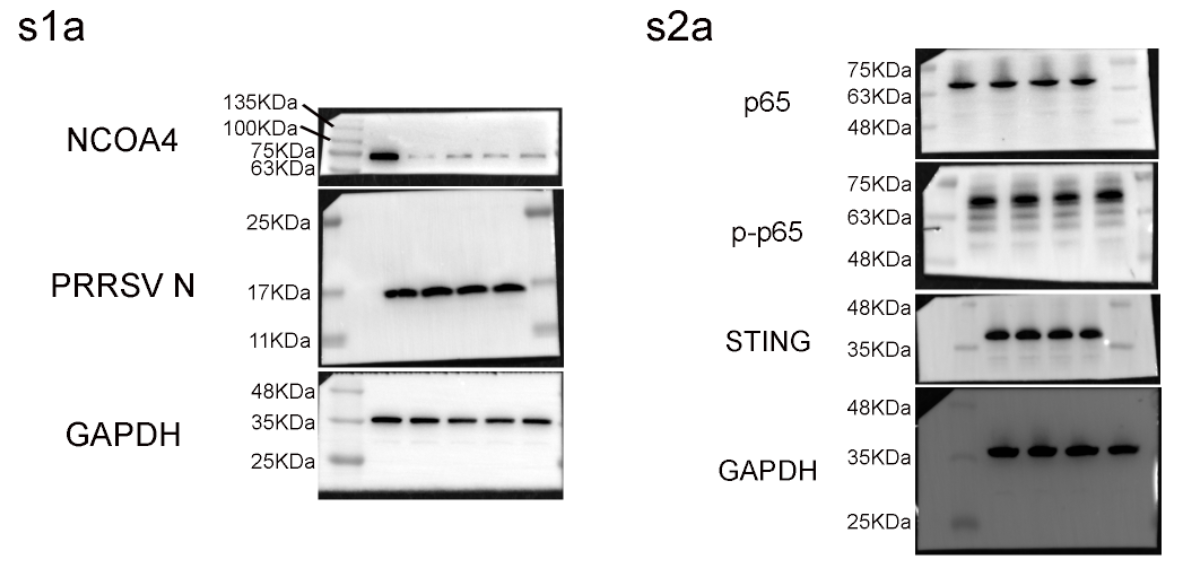


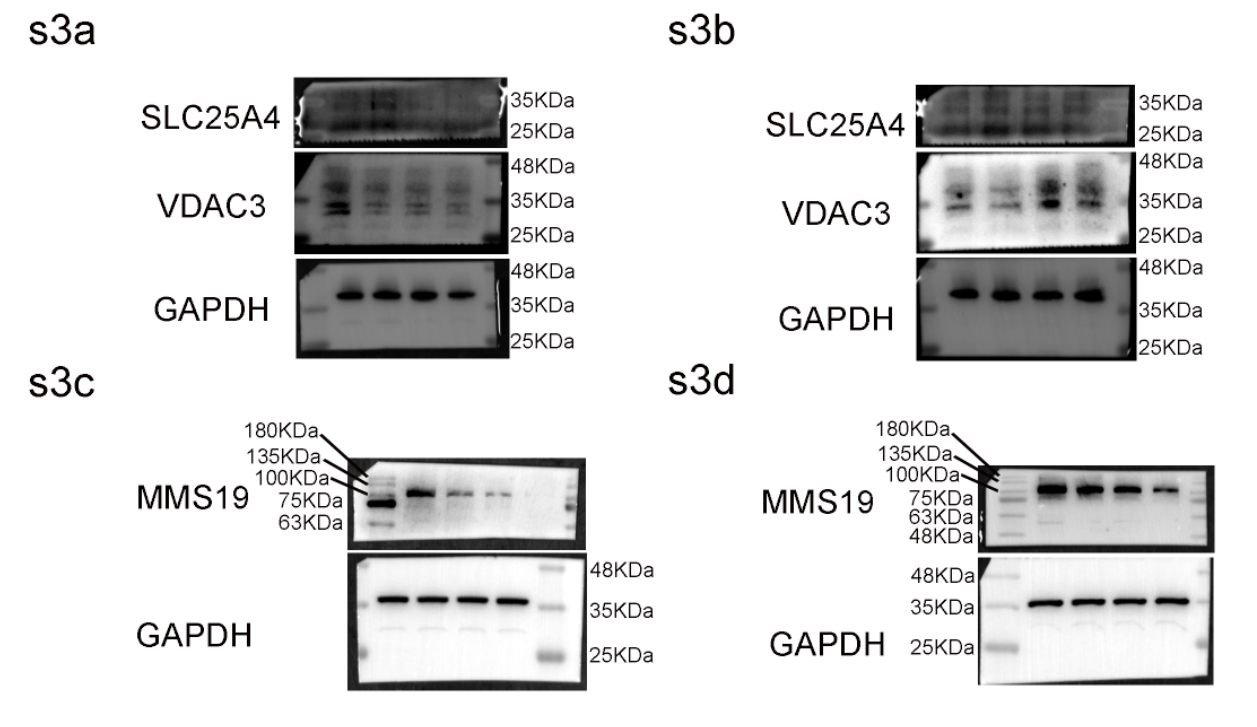


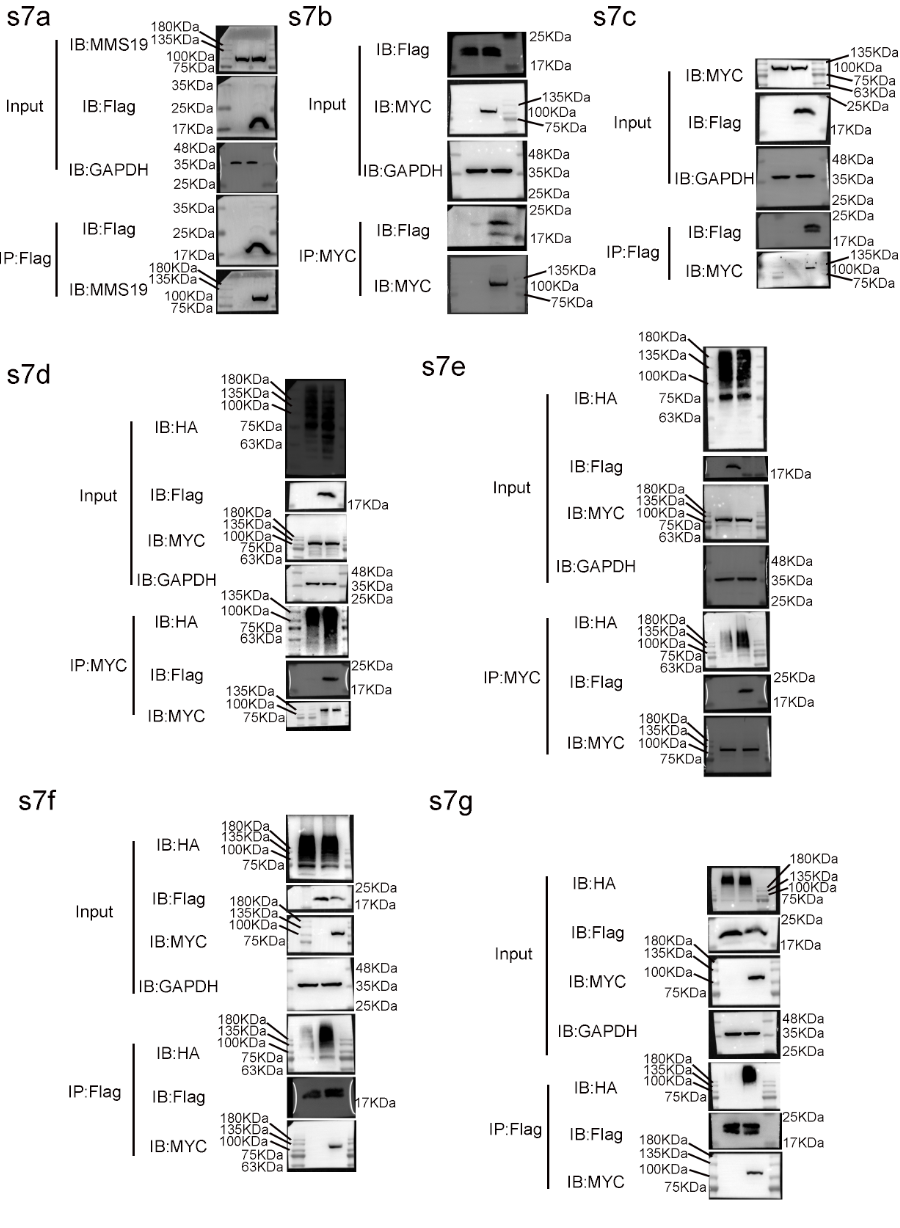

Supplement: Supplementary file 2 — Supporting File 2: advs75721‐sup‐0002‐FigureS9.docx. [file ADVS-9999-e75721-s003.docx]
